# VEGF Signal Complexity Confers Resistance to Atezolizumab, Bevacizumab, Carboplatin, and Paclitaxel in EGFR‐Tyrosine Kinase Inhibitor‐Resistant Non‐Small Cell Lung Cancer

**DOI:** 10.1002/mco2.70335

**Published:** 2025-08-19

**Authors:** Sehwa Hong, Namhee Yu, Ju Young Cho, Geon Kook Lee, Beung‐Chul Ahn, Youngjoo Lee, Hanna Sim, Bo Ram Song, Mihwa Hwang, Sunshin Kim, Jung‐Hyun Kim, Charny Park, Ji‐Youn Han

**Affiliations:** ^1^ Research Institute National Cancer Center Gyeonggi Republic of Korea; ^2^ Center for Lung Cancer National Cancer Center Hospital Gyeonggi‐do Republic of Korea; ^3^ National Cancer Center Graduate School of Cancer Science and Policy Gyeonggi Republic of Korea

**Keywords:** ABCP therapy response, combination immunotherapy, non‐small cell lung cancer, single‐cell transcriptome analysis, VEGF signaling

## Abstract

Atezolizumab, bevacizumab, carboplatin, and paclitaxel (ABCP) therapy is beneficial for epidermal growth factor receptor‐tyrosine kinase inhibitor (EGFR‐TKI)‐resistant non‐small cell lung cancer (NSCLC); however, the resistance mechanisms are not fully understood. In this study, we conducted a single‐cell RNA‐sequencing analysis of EGFR‐TKI‐resistant NSCLC patients grouped into ABCP responders and non‐responders. *VEGFA* was overexpressed in ABCP responders, whereas *VEGFC* was upregulated in non‐responders. VEGFA and VEGFC had exclusive distributions and interactions, suggesting their distinct roles. VEGFA facilitated the proliferation of responder tumor subcluster cells, whereas VEGFC secreted from non‐responder tumor cells interacted with tumor microenvironment cells. VEGFC predominantly cooperated with drug resistance pathways such as fibroblast growth factor signaling and YAP‐TAZ regulation, whereas VEGFA coordinated several oncogenic signaling pathways. *VEGFC* expression was the most significant prognostic marker (hazard ratio, 1.8 [95% confidence interval, 1.1–3.0], *p* = 0.015). Both VEGFA and VEGFC inhibition effectively suppressed tumor growth, suggesting that VEGF signaling complexity hampers the response to ABCP. In conclusion, combinatorial targeting of both ligands (VEGFA and VEGFC) or their receptors (VEGFR2 and KDR) may enhance the clinical benefit of ABCP in EGFR‐TKI‐resistant NSCLC patients.

## Graphical abstract

Single‐cell RNA sequencing of EGFR‐TKI‐resistant NSCLC reveals distinct roles of VEGFA and VEGFC in ABCP therapy response. VEGFA drives tumor proliferation in responders, while VEGFC promotes resistance through TME interaction, FGF signaling, and YAP‐TAZ regulation. Dual inhibition of VEGFA/VEGFC suppresses tumor growth, suggesting that combined VEGF targeting may enhance efficacy of ABCP in resistant NSCLC patients.

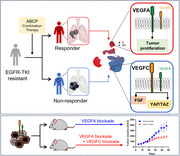

## 1 Introduction

Despite the significant improvement in survival with epidermal growth factor receptor (EGFR)‐tyrosine kinase inhibitors (TKIs), most patients with EGFR‐mutant non‐small cell lung cancer (NSCLC) develop EGFR‐TKI resistance. Therefore, additional therapeutic options following first‐line TKI treatment are urgently needed [1]. NSCLC tumor cells and the tumor microenvironment (TME) have distinct cellular characteristics. The TME of TKI‐resistant tumors plays a critical role in tumor cell proliferation and anti‐apoptosis. As tumors progress, immunosuppressive T cells, including regulatory and dysfunctional T cells, become predominant [2]. Cancer‐associated fibroblasts (CAFs) interact with tumor cells and secrete cytokines to suppress immune function, shaping a drug‐resistant microenvironment [3, 4]. Despite efforts to overcome EGFR‐TKI resistance, establishing a successful treatment for EGFR‐TKI‐resistant NSCLC remains challenging.

The Impower150 study aimed at addressing TKI resistance demonstrated a significant survival benefit of a combination of atezolizumab, bevacizumab, carboplatin, and paclitaxel (ABCP) over other immune checkpoint inhibitors combined with chemotherapy [1, 5]. Bevacizumab, a monoclonal antibody against vascular endothelial growth factor (VEGF)A, improved the response to ABCP compared to immunotherapy or chemotherapy and conferred a therapeutic benefit in EGFR‐TKI‐resistant NSCLC [6]. The upregulation of EGFR signaling may drive VEGF signaling in EGFR‐mutant NSCLC cells, contributing to TKI resistance [6]. VEGFs secreted from tumor and stromal cells affect regulatory T‐cell functions and desmoplastic stroma development [7]. VEGF family proteins contributing to tumor cell survival exhibit distinct biological activities depending on the cell type [8, 9, 10, 11]. Therefore, the superior outcomes of ABCP in EGFR‐mutant NSCLC may be owing to increased sensitivity to bevacizumab, which effectively blocks VEGF‐mediated oncogenic signaling activation or reverses immune suppression [6]. A randomized phase III trial demonstrated a significant progression‐free survival benefit of ABCP compared to pemetrexed plus platinum combination chemotherapy [12]. However, there were no significant differences in overall survival and response duration (median 7.1 months), and 50% of patients developed disease progression within 9 months [13]. A comprehensive understanding of the resistance mechanisms to ABCP would allow the development of a more efficient subsequent treatment.

Single‐cell RNA sequencing (scRNA‐seq) has provided valuable insights into the intricate interactions within the TME of EGFR‐TKI‐resistant NSCLC, particularly regarding the resistance mechanisms to combination therapies like atezolizumab and bevacizumab. These therapies target distinct aspects of the immune and vasculature systems, yet the complexity of the TME complicates their effectiveness. Single‐cell analyses of the TME have revealed significant cell heterogeneity, particularly the dominance of immunosuppressive T cells, fibroblast, and vascular development, in EGFR‐TKI‐resistant tumors. These immune cells contribute to immune evasion, undermining the efficacy of atezolizumab and bevacizumab [14, 15]. Moreover, CAFs contribute to immune exclusion and resistance to immunotherapy, and targeting these CAFs may enhance the efficacy of immune checkpoint blockade therapies [16]. The presence of hypoxic conditions also exacerbates the immune suppression [17]. Understanding these mechanisms at the single‐cell level provides crucial insights into why some patients respond better to ABCP therapy while others develop resistance, paving the way for more tailored therapeutic strategies.

**FIGURE 1:**
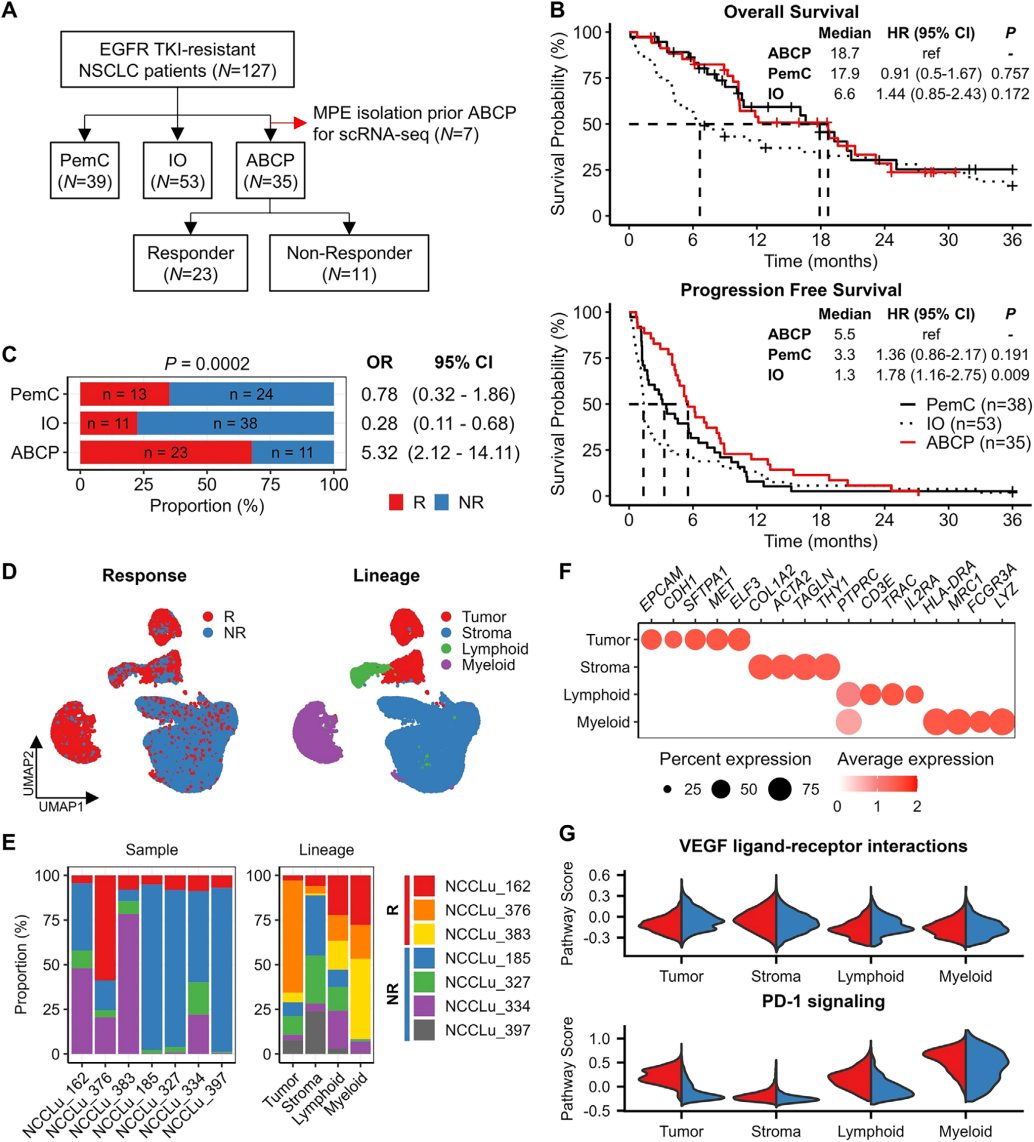
Combination immunotherapy (atezolizumab plus bevacizumab plus cisplatin and paclitaxel, ABCP) benefit and single‐cell transcriptome analysis. ABCP indicates the combination therapy, and PemC indicates pemetrexed. IO indicates immune oncology drugs. ABCP responder (R), red; non‐responder (NR), blue. (A) CONSORT flow diagram for NSCLC. (B) Overall survival and progression‐free survival plots according to the three therapy types. (C) Bar plot of therapy response percentage (R: responder, red, NR: non‐responder, blue) and OR with its 95% confidence interval (CI). (D) UMAP plots of the single‐cell transcriptome profile of seven patients, colored by ABCP responses or cell lineages. (E) Bar plots of cell proportions for each patient and cell lineage. (F) Heatmap of canonical markers to identify cell types. The circle size indicates the percentage of expressed cells, and the color indicates the average gene expression. (G) Activation status violin plots of ABCP target pathways for each cell lineage according to ABCP responses.

We aimed to elucidate the resistance mechanisms to ABCP further by conducting a scRNA‐seq analysis of EGFR‐TKI‐resistant NSCLC patients grouped into ABCP responders and nonresponders (Figure 1A). To identify ABCP response‐predictive markers, samples were acquired prior to ABCP therapy. To characterize superior responses to ABCP, we extracted biomarkers and identified the regulatory mechanisms. Our findings were validated using multiple platforms, including spatial scRNA‐seq, additional TKI‐resistant scRNA‐seq profiling, bulk transcriptome profiling, and immunohistochemistry (IHC) as well as both in vitro and in vivo experiments.

## 2 Results

### 2.1 Clinical Characteristics and Cell Distributions Prior to ABCP in TKI‐Progressed Patients

A total of 127 patients with EGFR‐mutant NSCLC progressed on prior EGFR‐TKI and received salvage treatment—ABCP arm: *n* = 35; immune oncology drugs (IO) arm: *n* = 53; pemetrexed and carboplatin (PemC) arm: *n* = 39—were analyzed for clinical efficacy (Table 1S, Figure 1A). EGFR mutations (Table S1, 85.7%–89.7%) were successfully inhibited by TKI (2.6%) to acquire the resistance. EGFR‐independent resistant genetic factors were observed (MET 5.7%–23.1% and other oncogenes KRAS, BRAF, and PIK3CA 2.6%–5.7%) [18]. These factors showed no association with ABCP response. ABCP treatment improved progression‐free survival compared to single immunotherapy (*p* = 0.009), and overall survival exhibited no dramatic outcome (*p* = 0.172; Figure 1B). More than 60% of patients responded to ABCP (*p* < 0.001, odds ratio [OR] = 5.32; Figure 1C), whereas approximately 40% did not. To identify the mechanisms and key predictive biomarkers of ABCP responsiveness, we conducted single‐cell transcriptome profiling of seven samples established before ABCP treatment using patient‐derived cells (PDCs) collected from malignant pleural effusions (responders, *n* = 3 and non‐responders, *n* = 4; Table S2, Figure 1A,D). In a previous study, it was already evaluated that the transcriptomic characteristics of these PDCs resemble those of tumor biopsy samples [19]. In total, 43,647 cells were isolated from live cell preparations with total counts ranging from 230,000 to 1,000,000 and viabilities from 55.3% to 90.5% for scRNA‐seq analysis. Next, these cells were clustered into four major cell lineages: tumor, stroma, lymphoid, and myeloid (Figure 1D). Stromal cells were enriched in non‐responders (OR = 29.09), whereas tumor (OR = 5.79) and myeloid (OR = 34.71) cells were more prevalent in responders (Figure 1E). Lymphoid cells constituted a small fraction (4.44%) of total cells. Cell clusters were annotated using canonical markers (Figure 1F, Table S3).

Overexpression of *EPCAM*, *CDH1*, *SFTPA1*, *MET*, and *ELF3* indicated MET‐dependent malignancies originating from epithelial cells (Figure 1F). The stromal cluster exhibited expression of fibrosis markers such as *COL1A2*, *ACTA2*, *TAGLN*, and *THY1*, whereas lymphoid cells expressed *PTPRC*, *CD3E*, and *TRAC*. Global ABCP target pathway assessment revealed higher PD‐1 signaling activation in responders than in non‐responders in both tumor (fold change [FC] = 1.98) and lymphoid (FC = 1.46) cells (Figure 1G). VEGF signaling did not differ between the two response groups. Although MPE samples are less heterogeneous than solid tumor biopsies, our analysis showed distinct differences between tumor and stromal cells that correlated with ABCP responsiveness [20].

### 2.2 Regulatory Programs and Therapeutic Responses Based on Tumor Cell Clustering

**FIGURE 2:**
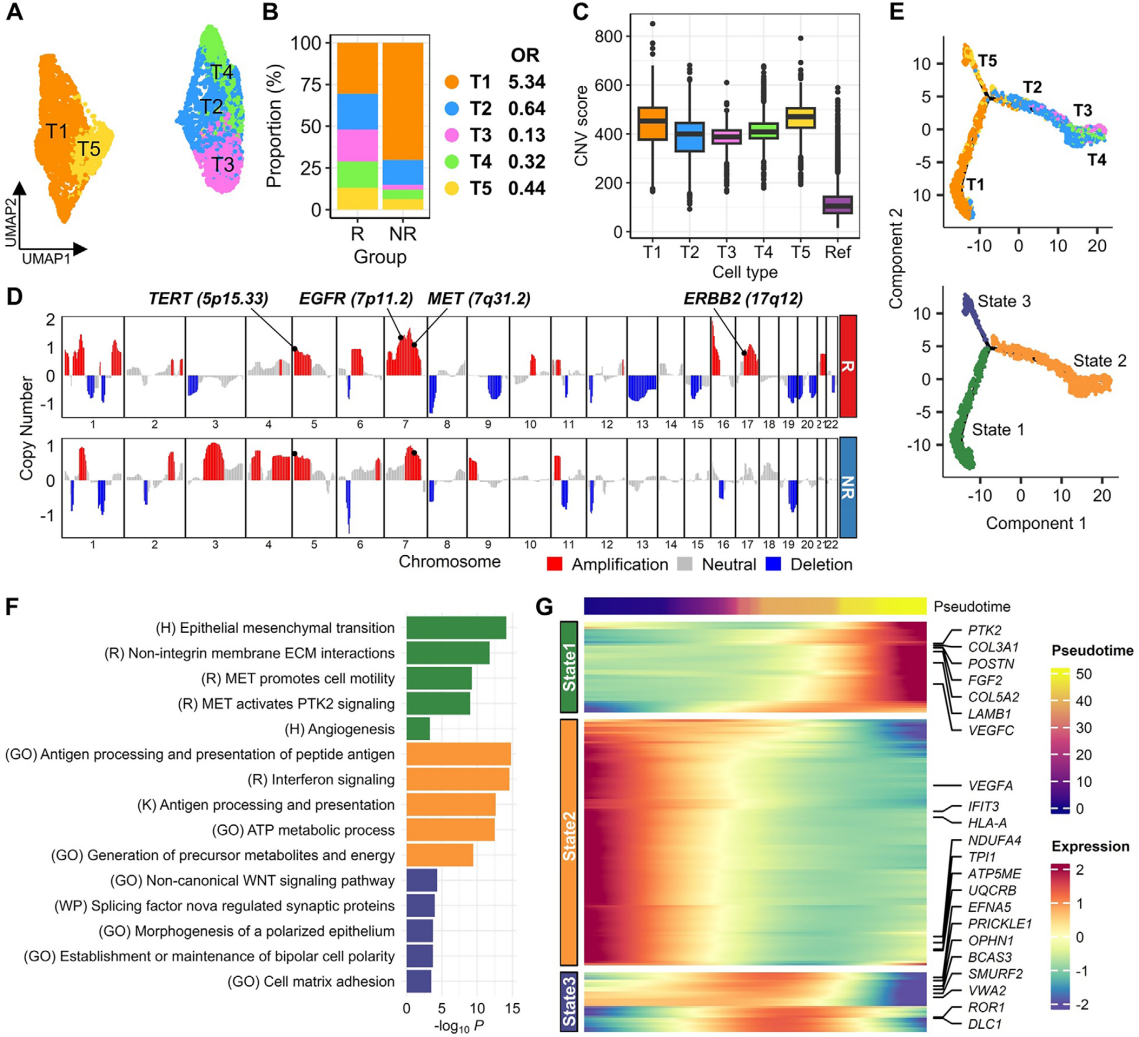
Tumor cell subclusters and their molecular mechanisms. R, ABCP responder; NR, non‐responder. (A) UMAP of tumor cells divided into five subclusters. (B) Cell proportion bar plot of five subclusters according to ABCP responses and ORs for each cluster. (C) CNV plots (*Y*‐axis) for all chromosomes (*X*‐axis) inferred from single‐cell transcriptomes and classified by ABCP response. Amplification gene names are shown, along with their locus. (D) Box plot of CNV scores for five tumor subclusters and the reference cell type (ref; myeloid). (E) Two dot plots of trajectory trees projected by two components of pseudo‐time. Dots are colored by cell types (left) or trajectory states (right). (F) Bar plot of *p*‐values for top‐ranked pathways extracted by GSEA using DEG sets of three states. (G) Heatmap of DEGs according to pseudo‐time (*X*‐axis) for three trajectory states (*Y*‐axis).

Tumor cell subclusters were associated with distinct oncogenic mechanisms. We identified five tumor subclusters with unique marker expression (T1–T5; Figure 2A,B, Figure S1A). Prior to characterizing the tumor cells, we assessed their malignancy. Overall, tumor cells had significantly higher copy number variation (CNV) scores than reference normal cells (Figure 2C). Amplifications in 5p15.33 (*TERT*) and 7q31.2 (*MET*) have been previously found to characterize lung adenocarcinoma (LUAD) profiles (Figure 2D) [21]. CNV analysis uncovered TKI‐induced resistance mechanisms. When categorized by therapeutic groups, amplifications of *TERT*, *EGFR*, *MET*, and *ERBB2* were observed in ABCP responders (Figure 2D). *TERT* and *MET* amplifications persisted in non‐responders. *MET* and *ERBB2* amplifications are established mechanisms of TKI resistance [18]. These CNVs indicated that these genes are potential therapeutic candidates.

To further demonstrate their malignancy, we compared the malignant cells identified in this study with all epithelial lineages in single‐cell transcriptome profiles of surgically resected LUAD samples (GSE131907) classified into tS1 (mixed), tS2 (tumor), and tS3 (normal) states based on their epithelial state [22]. We assessed the similarity between our data and the GSE131907 data after eliminating batch effects (Figure S1B). The five clusters in the present study were clearly dissimilar from normal epithelial cells (tS3). Subclusters T2–T4 exhibited the highest similarity with tumor cells (tS2). Clusters T1 and T5 had the highest CNV scores, indicating malignancy. Therefore, the tumor cluster in this study comprised malignant cells.

The five tumor cell clusters evolved into three distinct branches (States 1–3; Figure 2E). T1, predominantly associated with non‐responders (OR = 5.34; Figure 2B), progressed to State 1. T2–T5, primarily found in responders (OR < 0.64), branched into State 2 (T2–T4) or State 3 (T5). State 1 was characterized by activation of EMT, MET signaling, and angiogenesis pathways involving *FGF2*, *PTK2*, and *VEGFC* (Figure 2F–G). State 2 was associated with the upregulation of antigen‐processing and interferon signaling pathways and maintained proliferation via VEGFA. The State 3 branch, T5, demonstrated regulation of Wnt signaling and cell matrix adhesion involving *BCAS3*, *SMURF2*, and *ROR1*.

### 2.3 TME Involvement in the Response to ABCP Therapy

**FIGURE 3:**
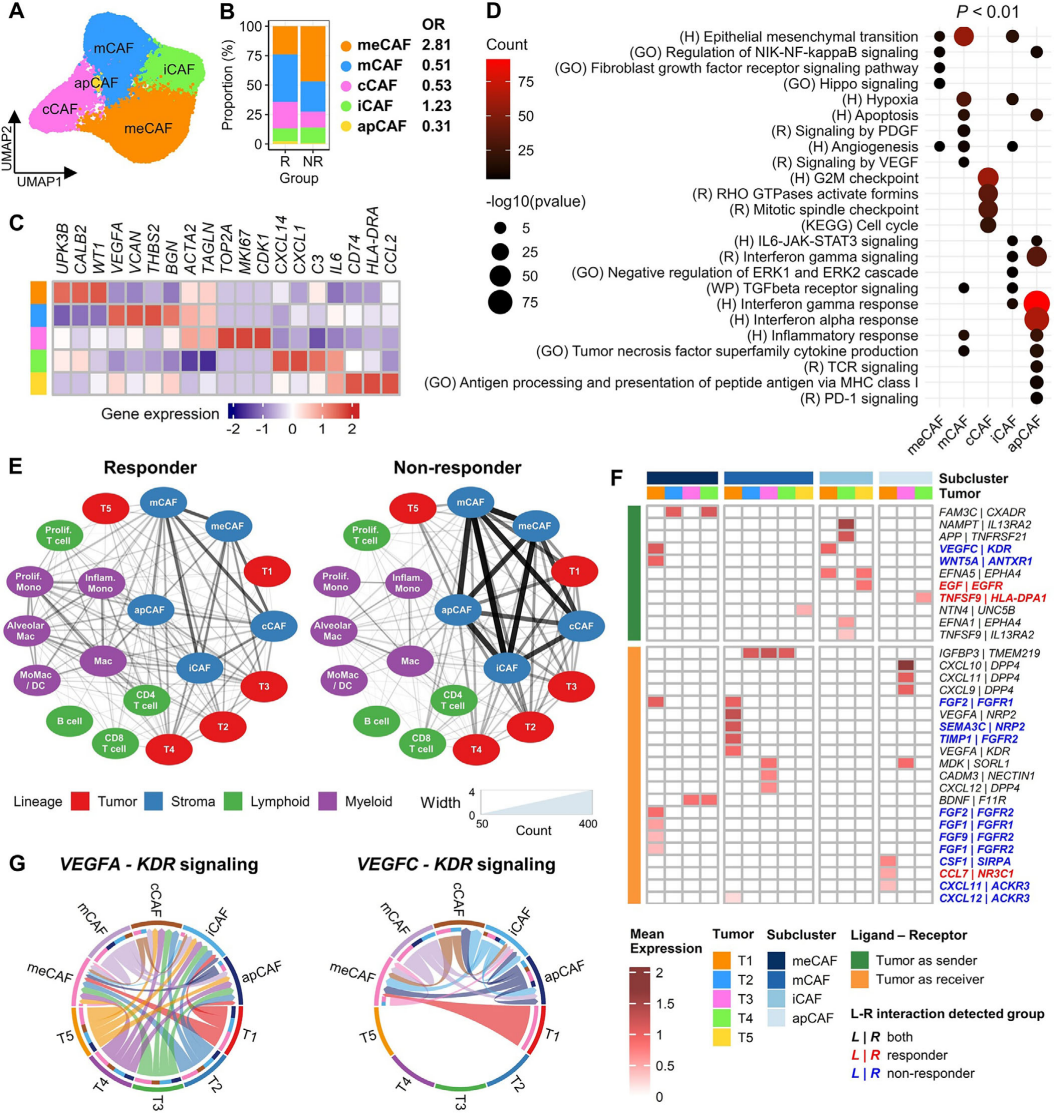
Stromal cell subclusters and their molecular mechanisms of cell–cell interactions. (A) Stromal cell subtype identification. UMAP of stromal cells, proportion bar plot of these cell types, and heatmap of canonical markers. CAFs were classified into five types: meCAFs (mesenchymal), mCAFs (myelofibrotic), cCAFs (cycling), iCAFs (inflammatory), and apCAFs (antigen‐presenting). These subtypes are indicated in five distinct colors. (B) Bar plot of pathway *p*‐values assessed by GSEA using differentially expressed genes for five CAF types. The circle size indicates the log‐scale *p*‐value, and the color indicates the number of detected genes. (C) Two cell–cell interaction networks for ABCP response groups. Nodes indicate cell types, and the edge width denotes the scale of cell–cell interaction counts. (D) Heatmap of cell–cell interactions for ligand–receptor pairing tumor subclusters with CAF subclusters. Interactions detected in the responder and non‐responder groups are denoted in red and blue, respectively. Signaling directions are colored differently on the left panel (green: tumor sender, yellow: tumor receiver). (G) *VEGFA*–KDR and *VEGFC*–*KDR* interaction between the tumor and CAF subcluster.

Stromal cells were classified into five subclusters, four of which corresponded to CAF types previously established based on canonical markers: myofibroblastic (mCAFs), cycling (cCAFs), inflammatory (iCAFs), and antigen‐presenting (apCAFs) (Figure 3A,B) [23]. We identified a novel subcluster, mesothelial CAFs (meCAFs) (expressing *UPK3B*, *CALB2*, and *WT1*, Figure 3C). This subtype exhibited EMT reprogramming and resembled CAF subtypes identified in scRNA‐seq profiles of MPE samples from triple‐negative breast cancer [20]. Common CAF subtype markers included extracellular matrix‐remodeling genes (*COL1A2*, *ACTA2*, and *TAGLN*), although each subtype exhibited distinct fibrotic programs. meCAFs showed FGF and Hippo signaling activation (Figure 3D). In contrast, mCAFs (*VEGFA*, *VCAN*, and *THBS2*) exhibited PDGF, VEGF, and angiogenic signaling activation.

Although the other three CAF types comprised smaller fractions (< 30%), subcluster identification revealed their main functional characteristics: cell cycle and Rho GTPases in cCAFs; IL6‐JAK‐Stat3, transforming growth factor‐beta (TGF‐β) receptor, and interferon‐gamma signaling in iCAFs; and antigen‐processing and T‐cell receptor signaling in apCAFs (Figure 3D). meCAFs were enriched in ABCP non‐responders (OR = 2.81), and mCAFs in ABCP responders (OR = 0.51; Figure 3B). Our findings align with previous classifications of three main CAF subtypes involved in activating tumor protection programs via FGF, PDGF, and TGF‐β signaling [24]. *VEGFA*‐*NRP2* interactions between mCAFs and T1 cell types were more frequently observed in ABCP responders (Figure 3E,F). Meanwhile, tumor cell type T1 sent signals through *VEGFC*‐*KDR* interactions to meCAFs and iCAFs. VEGF signaling consists of complex interactions to maintain tumor proliferation or to facilitate TMEs. ABCP combination therapy seems to effectively target VEGFA‐mediated signaling received by tumor cells from CAF subtypes.

Lymphoid and myeloid cells represented a relatively small fraction (21.71%) (Figure 1D,E), which is nearly half the fraction reported in a previous MPE single‐cell transcriptome profiling study [11]. Myeloid cells, primarily found in responders, were divided into five subclusters: alveolar macrophages, macrophages, inflammatory monocytes, proliferating monocytes, and dendritic cells (Figure S2A). Dendritic cells were more prevalent in non‐responders than in responders, although their population was small. Lymphoid cells were categorized into CD8^+^ T cells, CD4^+^ T cells, proliferating T cells, and B cells, which were evenly distributed between responders and nonresponders (Figure S2B). PD‐1 signaling was activated across CD4^+^ T cells and B cells. Our lymphoid profile was insufficient to resolve the specific cell subtypes or to infer their association with ABCP response. Subclustering analysis of tumor‐associated macrophages (TAM) identified three distinct subtypes: *SPP1* ^+^ TAM, M1 macrophages, and M2 macrophages (Figure S2C). M2 macrophages were predominantly derived from non‐responders (OR = 9.43), whereas M1 macrophages were enriched in responders (OR = 2.31). Our results provide evidence that M2 TAM contributes to PD‐1 blockade resistance by T‐cell exclusion from TME [25].

### 2.4 Cell–Cell Communication Emerges as a Therapeutic Vulnerability

To assess global cell–cell communication, we extrapolated interactions among all subclusters assorted according to the ABCP response and cell type. Interestingly, the cell interaction network divided into ABCP responses revealed distinct communication patterns (Figure 3E–G). In responders, mCAFs exhibited extensive interactions with meCAFs, cCAFs, and iCAFs and T2 and T3 tumor cells, whereas in non‐responders, all five CAF subtypes communicated intensively with each other and with T1 tumor cells (Figure 3E).

When analyzing ligand–receptor interactions, we identified subcluster‐specific and response‐specific interactions (Figure 3F). Tumor–stroma interactions were the most prevalent in both responders and non‐responders. Notably, in non‐responders, T1 tumor cells were engaged in an interplay with meCAFs via VEGFC–KDR signaling (kinase insert domain receptor; VEGF receptor 2) and exclusively received FGF2–FGFR1 signals from meCAFs (Figure 3F). Additionally, CXCL11/CXCL12–ACKR3 interactions in apCAFs promoted tumor progression in non‐responders [26].

Responder data revealed an interaction between epidermal growth factor (EGF) on T5 cells and EGFR on iCAFs, whereas TNFSF9 on T4 cells interacted with HLA‐DPA1 on apCAFs, which is favorable for immunotherapy [27]. CXCL9/CXCL10/CXCL11–DPP4 interactions, known to attenuate anticancer immunity, protect the T3 subtype [28]. Among angiogenesis regulators, the VEGFA–NRP2 signal, transmitted from mCAFs to T1 subtype cells, was commonly detected in both responders and non‐responders (Figure 3F). However, interactions between VEGFA or VEGFC and KDR displayed distinct patterns according to the cell type and ABCP response (Figure 3G). While VEGFA, expressed by all tumor cell types, facilitated tumor cell proliferation, the VEGFC–KDR signal was restricted to interactions between specific T1 tumor cells and other CAF subtypes, promoting the development of meCAFs. Our analysis implies that VEGF ligands could exert differential effects across cell types: VEGFA may be linked to tumor cell proliferation, while VEGFC appears to support CAF‐mediated tumor protection.

### 2.5 Spatial scRNA‐Seq Reveals Tumor Protection Resulting From ABCP Treatment

**FIGURE 4:**
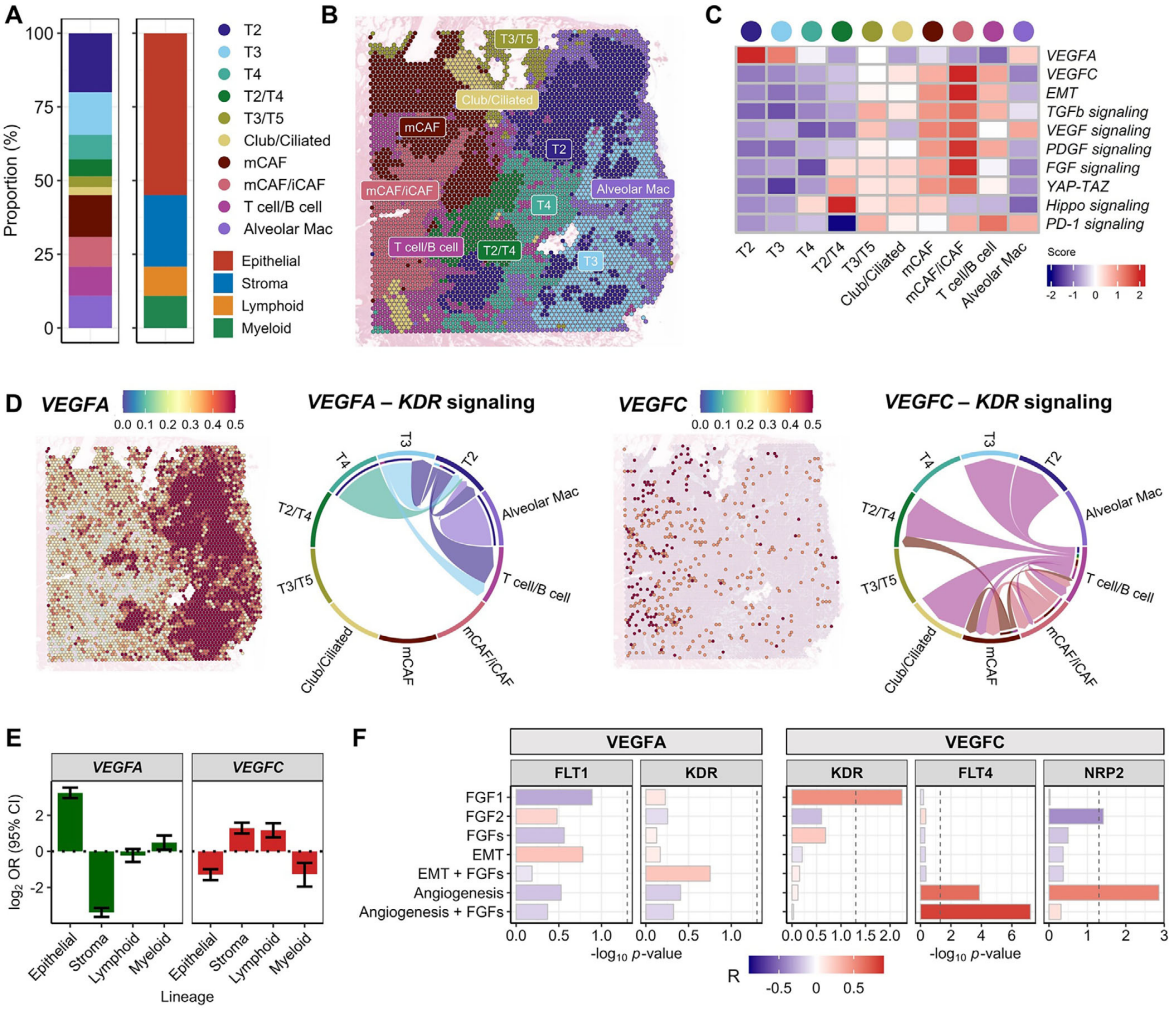
Spatial transcriptome profile of EGFR‐TKI‐resistant patient tissue. Capture area was 6.5 × 6.5 mm. (A) Two bar plots of cell proportions according to cell types and cell lineages identified from spatial transcriptome profiles. (B) Spatial cell type distribution. (C) Heatmap of gene expression and pathway scores for each cell type. (D) Spatial expression of VEGFA and VEGFC on a spatial tissue biopsy. Circular plots for VEGFA–KDR and VEGFC–KDR interactions between two cell types. (E) Two bar plot ORs between cell lineages and gene expression (*VEGFA* and *VEGFC*). (F) Bar plots for correlation coefficients of VEGFA or VEGFC expression with stromal markers assessed from IHC of patient tumor biopsies (*n* = 29). Color indicates the *R* value. The *X*‐axis indicates the *p*‐value. The *Y*‐axis indicates IHC marker sets (FGFs: FGF1 and FGF2, EMT: CDH2 and VIM, angiogenesis: POSTIN).

To validate our observations, we generated spatially resolved single‐cell transcriptomic profiles using tumor samples obtained from TKI‐resistant patients (*n* = 2) who had progressed following second‐line osimertinib treatment. We detected 4518–4886 cell spots and assigned cell types to the spatial transcriptome profiles based on cell scoring referring to the above cell subclusters and known global LUAD cell types (Figure 4A,B, Figure S3A,D) [22]. The abundance of stromal cell populations (22.86%–24.30%) resembled that of other TKI‐resistant cells (fibroblasts and endothelial cells: > 20%; Figure 4A,B, Figure S3C) [2].

*VEGFA* and *VEGFC* were expressed in different locations. *VEGFA* was notably overexpressed in tumor subcluster T2 (Figure 4C–E, Figure S3E–G), whereas *VEGFC* expression was observed in mCAFs and iCAFs, alongside the activation of EMT, PD‐1 signaling, YAP/TAZ signaling, and other fibrosis pathways, except Hippo signaling. Globally, *VEGFA* expression was over‐expressed in epithelial lineages rather than stromal cells, whereas *VEGFC* was expressed in stromal and lymphoid cells (Figure 4E). To further demonstrate co‐localization, we performed IHC using tissues acquired from EGFR‐TKI‐resistant patients (*n* = 30). Co‐localization between VEGF signal and TME‐associated markers was assessed in tissues (FGF signal: FGF1/2, angiogenesis: POSTN, and EMT: CDH2 and VIM; Figure 4F). *VEGFC* and receptors *VEGFR3*/*NRP2* exhibited a strong positive correlation with angiogenesis (*R* = 0.57) and FGFs (*R* = 0.82). VEGFA expression globally exhibited no correlation with EMT (CDH2^+^ VIM^+^; *R* = 0.26) and FGF signal (*R* = 0.25). In summary, *VEGFA* and *VEGFC* were expressed in mutually exclusive cell regions in epithelial and CAF cells of our TKI‐resistant patients, respectively. The correlation between FGF and VEGFA showed a moderate negative correlation, whereas VEGFC strongly interacted with FGF‐activated CAF subtypes, with angiogenesis.

VEGFA–KDR interaction was observed between tumor cell subclusters and immune cell types (Figure 4D), whereas the VEGFC–KDR interaction signal was exclusive to CAF cells. Consistent results were obtained for another TKI‐resistant sample showing *VEGFA* overexpression in tumor cell types and mutual exclusiveness of VEGFA and VEGFC (Figure S3E). Taken together, these findings indicated that VEGFA in tumor cells appears to maintain tumor cell proliferation, whereas VEGFC in TME cells protects tumor cells via mCAF development with FGF signaling activation or YAP‐TAZ regulation.

### 2.6 Angiogenic and Fibroblastic Signals According to Therapeutic Status

**FIGURE 5:**
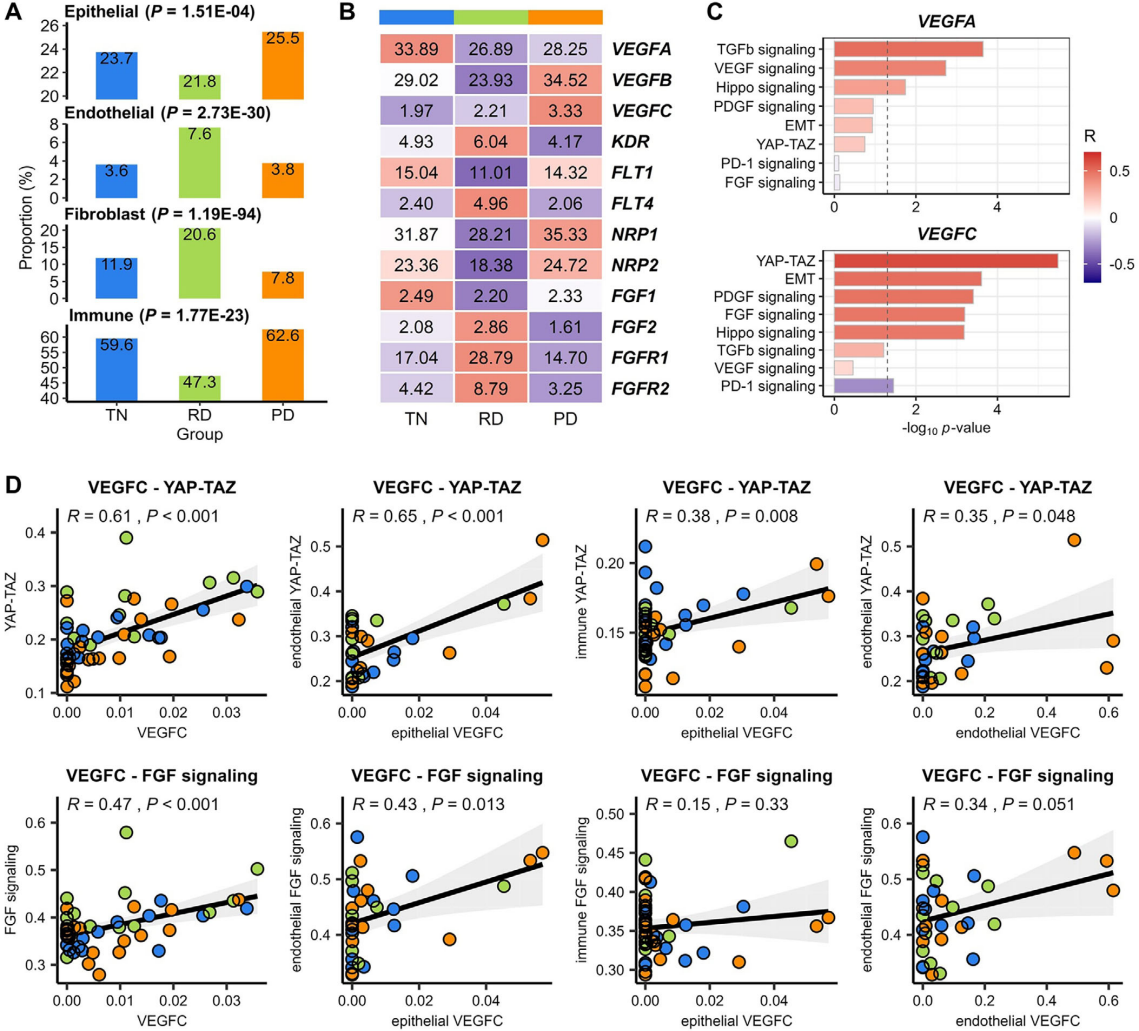
Demonstration of VEGF signal genes and cooperative pathways using therapy‐induced transcriptome dynamics. TKI treatment groups were classified into three types: TN: treatment‐naïve, blue; RD: residual disease, yellow‐green; PD: progressive disease, orange). (A) Bar plots of cell proportions for three therapy groups and four cell lineages. (B) Heatmap of average gene expression of VEGF and FGF signaling for treatment groups. (C) Correlation coefficient bar plots of stromal‐associated pathways with *VEGFA* and *VEGFC*. Bar color indicates correlation coefficients. The X‐axis indicates the log‐scale *p*‐value. (D) Scatter plots of *VEGFC* expression (X‐axis) and pathway scores (Y‐axis). Each spot is an expression value calculated by pseudo‐bulking for each patient, each cell type, and all cell types. Dots are colored by therapy groups.

To uncover the dynamic changes in angiogenic and fibroblastic signals according to therapeutic status, we used an scRNA‐seq dataset of dissected tumor biopsies (*n* = 49) from three TKI therapeutic groups: TKI‐naïve (TN), residual disease (RD) during treatment and stable status, and progressive disease (PD) [29]. As these samples were collected during disease progression following TKI treatment of the primary tumor, the target mechanisms of ABCP could be dissected in the context of stepwise resistance acquisition. Cell types were defined referring to the original report (Figure 5A). In RD, the endothelial cell and fibroblast populations were twice as abundant as others (Figure 5A), implying that angiogenic and fibroblastic development is amplified in RD. When examining the above markers identified in ABCP non‐responders, we found that *VEGFA* expression was upregulated in RD, *VEGFC* expression in PD, and VEGF receptor gene expression in RD (Figure 5B). *VEGFA* was upregulated by 1.103‐ to 1.110‐fold in RD and PD epithelial cells compared to TN (Figure S4). *VEGFC* expression showed a 6.04‐fold increase in PD epithelial cells compared to TN and RD, although its absolute expression level was lower than that of *VEGFA*. VEGF receptor gene expression showed a twofold increase in RD endothelial cells, whereas co‐receptor genes had the highest expression levels in PD epithelial cells. FGF signaling was activated, along with an increase in fibroblasts, in RD. In summary, VEGFA secreted from tumor cells exhibits predominant expression starting in the TN stage. Alternatively, VEGFC originating from fibroblasts induces tumor cell progression from TN to PD. Endothelial cell development via VEGF receptors is directly relevant to ongoing TKI treatment. Meanwhile, the co‐receptors NRP1 and NRP2 play cooperative roles in epithelial cells and fibroblasts during treatment and are co‐expressed with VEGF receptors in endothelial cells. This implies that the complexity of VEGF signal interaction increases after TKI treatment and progresses to dominant VEGFA regulation, supporting the need for diversified interventions to target interactions with TME cells.

To investigate cooperative functions with global *VEGFA* and *VEGFC* expression, we assessed correlations with the stromal pathways extracted from the ABCP cohort (Figure 5C). *VEGFC* expression showed predominantly positive correlations with five stromal pathways, particularly, YAP‐TAZ and EMT (*p* < 0.001), whereas TGF‐β was associated with *VEGFA* expression. We next decomposed VEGFC‐associated signals to cell types for YAP‐TAZ and FGF signaling (Figure 5D). In epithelial and endothelial cells, *VEGFC* expression stimulated the upregulation of YAP‐TAZ and in endothelial cells, it promoted FGF signaling activation (*p* < 0.05). Thus, the increase in *VEGFC* expression following therapeutic progression facilitates endothelial cell development via YAP‐TAZ regulation and FGF signaling activation.

### 2.7 Global Regulation Programs and Prognosis Governed by VEGFA and VEGFC

**FIGURE 6:**
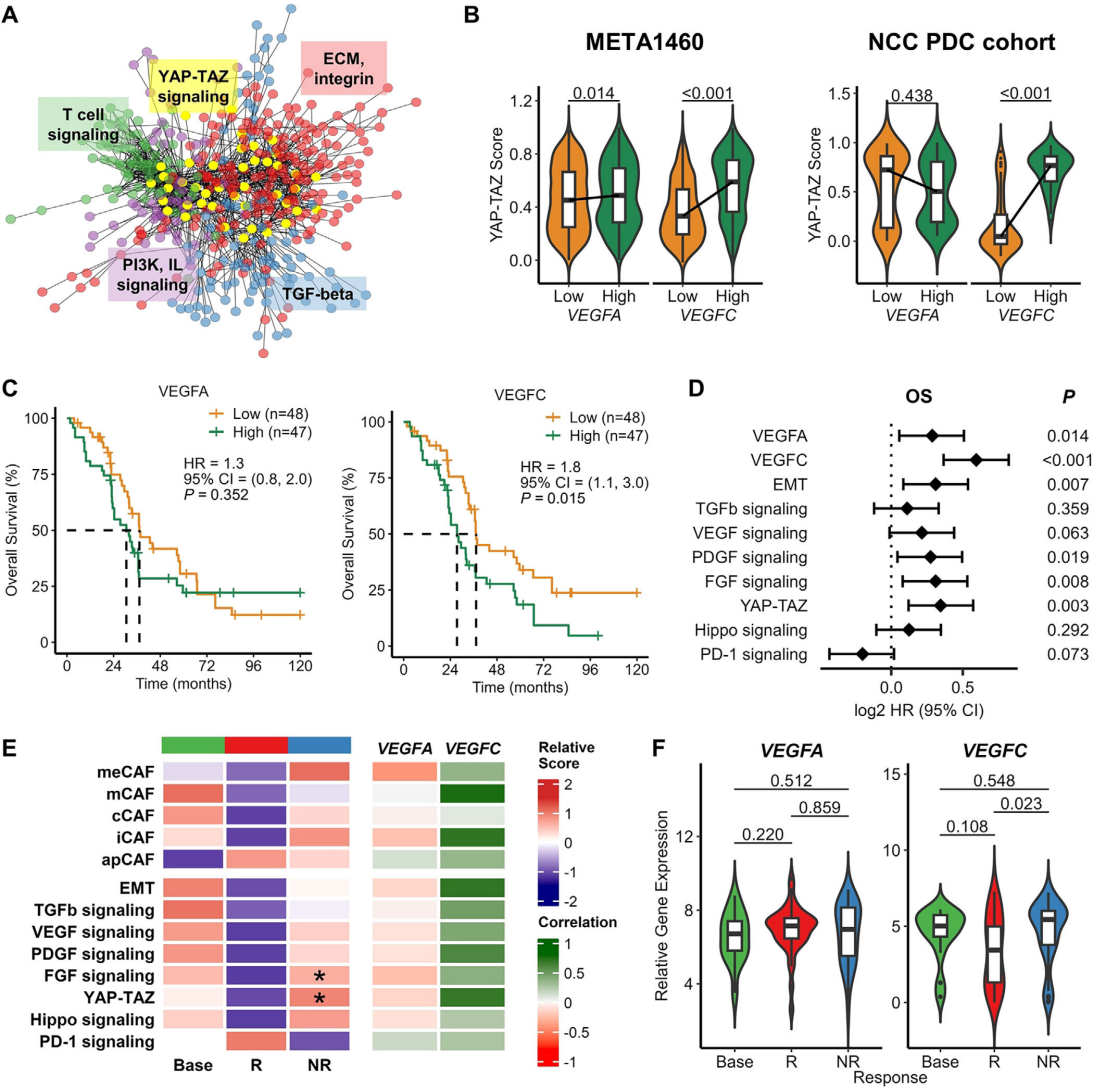
Meta‐transcriptome analysis of NSCLC reveals global regulatory programs of *VEGFA* and *VEGFC*. Samples were classified into two types by VEGF gene expression. The high group is the upper median, and the low group is the lower median grouped by *VEGFA* and *VEGFC* gene expression. EGFR‐TKI treatment profiles were categorized into three groups: base, baseline (green); responder, R (red); non‐responder, NR (blue). (A) Gene–gene interaction network regulated by VEGFC. Nodes are colored by network modules, and pathways are highlighted based on gene set enrichment analysis. (B) Violin plots for YAP‐TAZ score according to *VEGFA* and *VEGFC* gene expression for META1460 and NCC PDC cohort patients. (C) Survival plots according to *VEGFA* and *VEGFC* expression for overall survival of the NCC PDC cohort. (D) Forest plot for HR by overall survival according to VEGF gene and pathway scores of the META1460 profile. (E) Two heatmaps of scores and correlation coefficients. Left: heatmap for scores for cell types and pathways according to three groups. *p < 0.05 versus responders. Right: heatmap for correlation coefficients with *VEGFA* and *VEGFC* expression. (F) Violin plots for *VEGFA* and *VEGFC* gene expression according to EGFR‐TKI treatment groups.

To dissect the differential global regulatory programs governed by VEGFA and VEGFC, we conducted gene–gene interaction network analysis using bulk RNA‐seq transcriptome profiles (META1460, *n* = 1460) obtained from several NSCLC cohorts (Figure 6A, Table S4). We established co‐expression interaction networks regulated by VEGFA and VEGFC. Submodules of these networks were identified, and their regulatory pathways were investigated through gene set enrichment analysis. For further investigation of clinical profiles, we employed our National Cancer Center (NCC) PDC cohort (*n* = 95; Table S5) data acquired from MPE samples [19].

Network analysis identified common pathway modules between VEGFA and VEGFC involved in extracellular matrix organization, angiogenesis, VEGF signaling, and PDGF‐β signaling (Figure S5). We also found distinct modules, suggesting potential differences in cascading or cooperative signaling mechanisms employed by VEGFA and VEGFC. VEGFA was associated with oncogenic signals such as ERBB2, RAS, mTOR, and the p53 pathway [30], whereas VEGFC cooperated with TME development through pathways including TGF‐β, T‐cell activation, interleukin signaling, Rho GTPase activation, and Toll‐like receptor signaling (Figure 6A, Figure S5) [31]. *FGF1* and *FGF7* were detected in the VEGFC co‐expression network, in the TGF‐β and Toll‐like receptor modules, respectively. YAP‐TAZ upregulation by VEGFC was found in both the META1460 and NCC cohorts (VEGFC, *p* < 0.001; VEGFA, *p* = 0.014; Figure 6B). Overall, these co‐expression networks elucidated the distinct roles of the two VEGF ligands: VEGFA contributes to tumor proliferation, whereas VEGFC potentiates desmoplasia and anticancer immunity.

To assess the clinical outcomes associated with the expression of these genes, we performed survival analysis. In the refractory lung cancer NCC PDC cohort, *VEGFC* expression upregulation was significantly correlated with poor overall survival (*p* = 0.015, hazard ratio [HR] = 1.8, Figure 6C). In the NSCLC‐integrated cohort META1460, *VEGFC* expression was a stronger predictor of overall survival (*p* < 0.001; HR = 1.51) than other pathway markers (Figure 6D).

We additionally analyzed NCC PDC cohort EGFR‐TKI response profiles (EGFR‐TKI groups: baseline *n* = 15; responder *n* = 38; non‐responder *n* = 42) to characterize the therapy‐induced status of VEGFs and additional signatures (Figure 6E, Table S5). EGFR‐TKI non‐responders showed increased *VEGFC* expression (*p* = 0.023), whereas *VEGFA* remained consistent across all therapeutic statuses (Figure 6F). FGF, YAP‐TAZ signaling, and meCAF scores were elevated in non‐responders, as was *VEGFC* expression (*p* = 0.054). These cell and pathway scores were mostly correlated with *VEGFC* expression rather than *VEGFA* expression (Figure 6E). Particularly, EMT and YAP‐TAZ exhibited the strongest correlations (*R* = 0.691, *p* < 0.001). These findings suggested that, while a VEGFA‐targeted drug such as bevacizumab may be effective from baseline, TKI‐non‐responders have an increased risk of bevacizumab failure due to VEGFC in cooperation with FGF signaling and YAP‐TAZ regulation. Therefore, targeting VEGFC or blockade of its interactor KDR should be considered for TKI‐non‐responders.

### 2.8 Combinational Knockdown of *VEGFC* and Its Receptors Blocks Drug Resistance and Angiogenesis by Inhibiting FGF/YAP/TAZ

To elucidate the role of VEGFs/VEGF receptors in drug resistance, we conducted in vitro assays using lung cancer cell lines classified as sensitive or resistant based on VEGF/VEGF receptor (*VEGFC*, *KDR*, and *NRP2*) expression and IC_50_ values of anti‐VEGF drugs (cabozantinib, pazopanib, and sitravatinib). H1573, H2444, and H2279 cells were categorized as resistant, and H1581 and H1437 as sensitive (Figure S6A,B).

**FIGURE 7:**
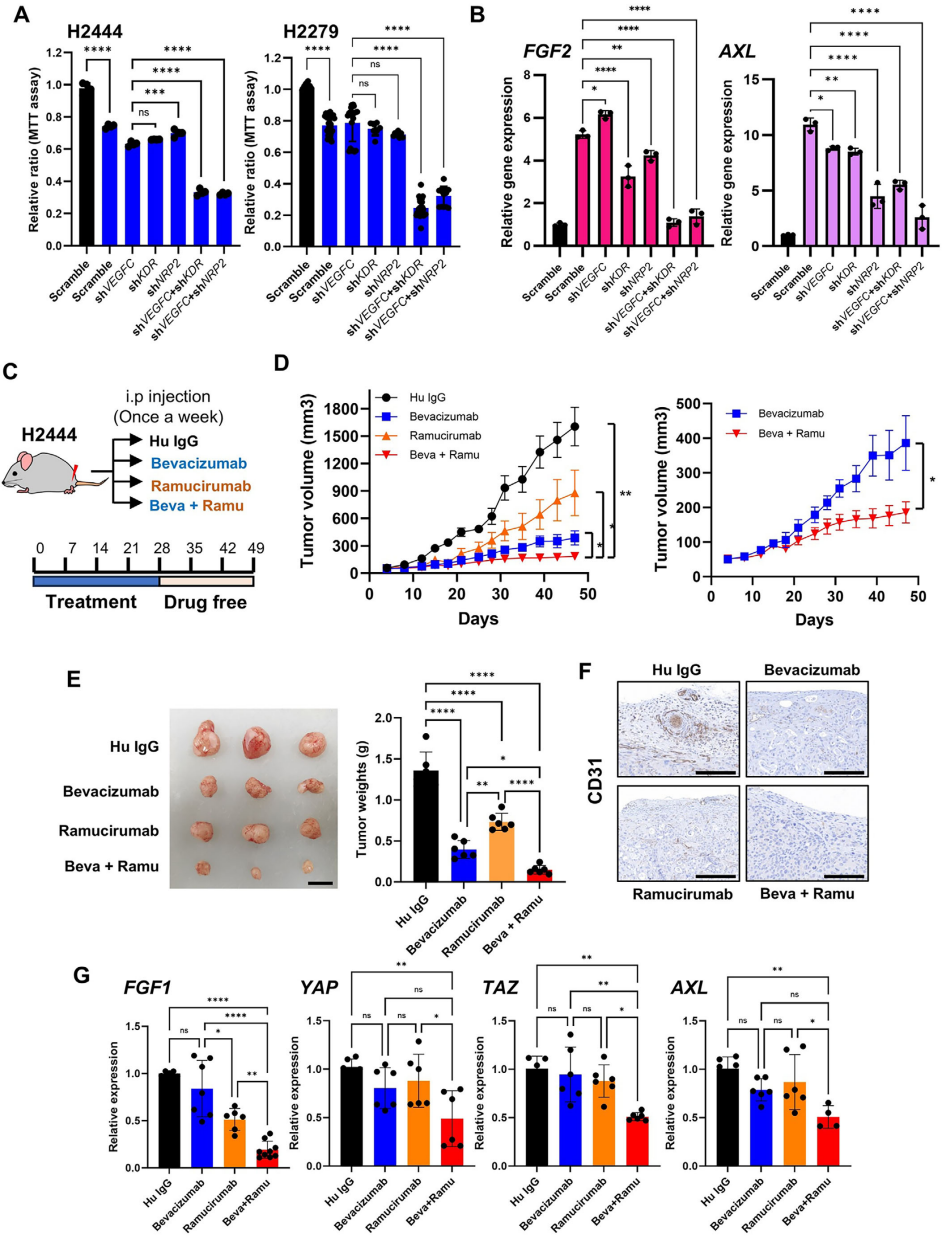
Inhibition of the VEGF pathway significantly enhances the anticancer effects of VEGF‐targeted drugs in both in vitro and in vivo conditions. (A) Bar graphs of the relative cell proliferation ratio following cabozantinib treatment with or without specific target gene knockdown in H2444 (left) and H2279 (right) cell lines. The black bar indicates DMSO treatment, and the blue bars indicate cabozantinib treatment (*n* = 3). (B) Bar graphs of qPCR results (*n* = 3) visualizing the expression of *FGF2* (left) and *AXL* (right) following target gene silencing with or without cabozantinib treatment (marked by color bars). (C) Experimental scheme outlining in vivo mouse experiments with various treatment options: human IgG (Control), bevacizumab (VEGFA‐targeted antibody), ramucirumab (KDR/VEGFR2 targeted antibody), and bevacizumab + ramucirumab. (D) Tumor growth curves of each treatment group. H2444 cells were subcutaneously implanted into flank of mice and treated accordingly. The left plot shows tumor growth across all four groups, while the right plot highlights only the bevacizumab‐treated group and bevacizumab + ramucirumab‐treated group for clear comparison between two groups. (E) Representative image of the tumor masses (left) and corresponding tumor weights (right) from H2444‐engrafted nude mice, scale bar = 1 cm. (F) Representative CD31‐stained IHC images from each tumor sample. Scale bar = 200 µm. (G) RT‐qPCR results (*n* = 6) for the expression of *FGF1, YAP, TAZ, AXL* in xenografted mouse tumor samples with various treatments. *p < 0.05, **p < 0.01, ***p < 0.001, ****p < 0.0001, one‐way ANOVA followed by Tukey's multiple comparison tests.

To assess whether VEGFC, KDR, and NRP2 are required for the anti‐VEGF drug response, we investigated the effects of cabozantinib on the proliferation of resistant cells with or without shRNA‐mediated depletion of *VEGFA*, *VEGFC*, *KDR*, and *NRP2*. Silencing of both *VEGFC* and *KDR* or *NRP2* significantly decreased cell proliferation compared to a scramble control or single‐gene silencing in the three resistant cell lines (Figure 7A and Figure S6C,D). However, the effect of *VEGFA* knockdown was not significantly stronger than that of *VEGFC* knockdown when co‐silenced with *KDR* or *NRP2* (Figure S6E). Two well‐characterized anti‐VEGF drugs, bevacizumab and ramucirumab, did not demonstrate combinational effects with *VEGFC/KDR/NRP2* silencing, as they did not affect cell proliferation in vitro, which is consistent with previous findings [32, 33].

We found a positive correlation between *VEGFC/KDR/NRP2* expression and the FGF pathway (associated with angiogenesis) and YAP/TAZ/AXL pathway (related to drug resistance) in non‐responders (Figure 3). As these pathways exhibit compensatory activation upon cabozantinib treatment in resistant cells [34], we investigated whether *VEGFC/KDR/NRP2* knockdown elicited a decrease in *FGF/YAP/TAZ/AXL* expression in resistant lung cancer cells. Combined knockdown of *VEGFC* and *KDR* or *NRP2* efficiently diminished cabozantinib‐mediated gene expression compared to scramble or single knockdown (Figure 7B and Figure S6F).

Next, we performed in vitro angiogenesis assays using HUVECs cultured in conditioned medium derived from lung cancer cells with or without shRNA. HUVECs cultured in conditioned medium from cells silenced for both *VEGFC* and *KDR* or *NRP2* exhibited decreased tube formation and tube length compared to HUVECs cultured in conditioned medium derived from control or single‐silenced cells (Figure S6G). These findings demonstrated that VEGFC–KDR and VEGFC–NRP2 play critical roles in drug resistance, angiogenesis, and related gene expression in drug‐resistant lung cancer cell lines.

### 2.9 KDR (VEGFR2) Blockade by Ramucirumab Enhances Sensitivity to Bevacizumab

To assess the antitumor efficacy of VEGFC–KDR blockade, we evaluated the efficacy of ramucirumab in H2444 cell‐engrafted nude mice. After subcutaneous injection, xenografted mice were randomly divided into four groups (*n* = 6 per group) and injected intraperitoneally with bevacizumab (2 mg/kg), ramucirumab (5 mg/kg), bevacizumab plus ramucirumab, or human IgG control (7 mg/kg) once a week (Figure 7C). Tumor growth and size were monitored. As expected, mice treated with human IgG exhibited the highest tumor growth rate, with the largest tumor size observed 49 days after H2444 cell injection. Single treatment with bevacizumab or ramucirumab reduced tumor growth and size. Recipients of H2444 cells with bevacizumab and ramucirumab showed the lowest tumor growth rate (Figure 7D,E) and size, without any loss in body weight (data not shown). Consistent with in vitro experimental results, cotreatment with bevacizumab and ramucirumab drastically diminished tumor angiogenesis, as evidenced by decreased CD31‐positive staining (Figure 7F), and suppressed the expression of angiogenesis and drug resistance‐related genes, *FGF* and *YAP/TAZ/AXL* (Figure 7G).

## 3 Discussion

Although ABCP treatment is a promising option for EGFR‐TKI resistant patients to improve PFS, the therapeutic mechanism of non‐responsiveness is still unclear. Single cell transcriptome analyses have revealed the characteristics of the response of NSCLC neoadjuvant immunotherapy combined with chemotherapy [35, 36, 37]. These studies demonstrated that TME populations, such as TAM, NK cells, or *CCR8* ^+^ regulatory T cells, are associated with therapeutic response. While these studies provide a comprehensive view of the TME landscape and its role in therapy, the mechanisms underlying responses related to VEGF signaling are not yet fully understood. Therefore, we assessed various transcriptomes from TKI‐resistant NSCLC and uncovered molecular features that disrupt the response to ABCP. Our results indicate that the VEGFC signal plays an important role in the development of fibroblasts regulated by the FGF signal and YAP/TAZ/AXL axis, as well as angiogenesis. Notably, VEGFC exhibited a stronger prognostic potential compared to VEGFA.

VEGF signaling network is a complex ligand–receptor system to play a role in angiogenesis, lymphangiogenesis, and vascular permeability [7]. VEGF ligands exhibit selective binding affinities: VEGFA primarily binds to VEGFR1/R2, whereas VEGFC/D predominantly bind to FLT4 (VEGFR3). Additionally, co‐receptor NRP1 enhances VEGFA‐VEGFR2 interaction, and NRP2 facilitates VEGFC/D interactions with their receptors. It implies that VEGFA inhibition alone could not perfectly block VEGF‐mediated intercellular communication due to alternative ligand and co‐receptor interactions. Here, we identified *VEGFA*‐expressing tumor cells in ABCP responders, which is consistent with previous LUAD studies [22, 38]. In ABCP non‐responders, *VEGFC* expression was substantially lower than *VEGFA* expression. Computational scores for VEGF signaling or angiogenesis pathways predominantly followed the expression trends of *VEGFA* and its receptors, showing increased abundance. To precisely assess the cell interaction complexity of VEGF signaling, we comprehensively investigated all ligand–receptor pairs using bulk transcriptome data. Interestingly, VEGFC showed no cell‐type‐specific expression across tumor cells and CAFs. However, the final phenotypic condition by VEGFC exhibited concurrent pathway activation and stromal development. *VEGFC* expression was significantly correlated with fibroblastic pathways, including FGF, TGF‐β, and PDGF‐β signaling. Increased FGF signaling is associated with resistance to anti‐VEGF agents [39]. VEGFC activates the canonical Hippo pathway in lymphatic endothelial cells, affecting YAP‐TAZ signaling [40]. In the tumor ecosystem, the biological functions of VEGFA and VEGFC are primarily linked to tumor angiogenesis and lymphangiogenesis. Our findings further emphasize the role of VEGFC in cooperating with fibroblastic regulation in tumor development and resistance, highlighting the specific mechanisms by which CAFs interact with VEGF signaling.

High tumoral VEGFC expression poses a higher risk in terms of survival than high VEGFA expression in NSCLC [41]. Moreover, VEGFC is a better biomarker for predicting poor outcomes than other representative fibroblastic regulators. Our NCC PDC refractory lung cancer cohort includes multiple TKI‐progressed patients, making it a valuable resource for developing ABCP strategies that consider TKI treatment status. Targeting VEGFA may be effective as an initial therapy, whereas targeting VEGFC would be more beneficial after the acquisition of TKI resistance.

While advancements in anti‐VEGF drugs such as bevacizumab have allowed targeted inhibition of VEGFA, these drugs have various side effects and trigger the upregulation of other factors promoting angiogenesis and drug resistance [42, 43]. VEGFC mediates glioblastoma survival, tumorigenicity, and bevacizumab resistance via KDR activation [44]. These findings suggest a potential role for VEGFC/KDR signaling in VEGF‐targeted drug resistance. Our scRNA‐seq analysis identified VEGFC/KDR as key molecules for ABCP resistance, and in vitro and in vivo models successfully revealed the resistance mechanisms of VEGFC/KDR to anti‐VEGF agents in NSCLC. Treatment with ramucirumab, a KDR‐targeting monoclonal antibody, in combination with bevacizumab efficiently reduced tumor growth and angiogenesis in an in vivo xenograft mouse model, suggesting a novel therapeutic strategy for drug‐resistant patients with NSCLC. Moreover, combined knockdown of *VEGFC* and its receptor (*KDR* or *NRP2*), targeting the VEGFC/KDR pathway, significantly enhanced the antitumor effects of cabozantinib in vitro and drastically suppressed the expression of cabozantinib‐induced drug resistance genes, *YAP/TAZ* and *FGF*. The YAP‐TAZ targets AXL and FGFs were upregulated in TKI‐resistant cells, and clinical trials and new drug developments for the inhibition of AXL and FGFs are ongoing [45, 46]. Therefore, developing combination therapies that inhibit FGF and YAP‐TAZ may further improve therapeutic success.

This study has several limitations. First, MPE samples exhibit less cellular heterogeneity than tumor biopsies [35, 36, 37]. The ABCP patient sample size was insufficient to clearly determine molecular features according to ABCP response. However, MPE can be used for tumor progression diagnosis without a biopsy burden, and we successfully uncovered the ABCP resistance mechanism from the limited samples. To overcome this limitation, we validated our findings using various sequencing platforms and a meta‐cohort of over 1000 samples. Second, the transcriptomic characteristics of our samples resembled those of tumor biopsies, but the reliability of interactions between tumor and TME cells may be limited. Therefore, we demonstrated concordance between our results and tumor tissues by performing cross validations using spatial scRNA‐seq and external tumor scRNA‐seq datasets. Furthermore, our samples exhibited an inadequate landscape of immune cells to infer the association with therapeutic response. Other immunotherapy studies could explore comprehensive mechanisms of anti‐PD1 therapy to cooperate with immune cells, including macrophages and T‐cell subtypes. In our analysis of macrophage subtypes, we observed that M2‐TAM showed less responsive to ABCP. However, the dynamics of macrophage polarization during immunotherapy and the complex crosstalk among the tumor and various immune cell types remain to be fully elucidated.

In summary, we profiled single‐cell transcriptomes of EGFR‐TKI‐resistant NSCLC patients to classify the molecular features determining the response to ABCP. Cross‐evaluations in multiple cohorts highlighted that VEGFC cooperates with fibroblastic mechanisms and promotes worse outcomes. Our results provide for potential combination therapy to overcome EGFR‐TKI resistance, and we anticipate an accelerated development of various anti‐VEGF agents.

## 4 Methods

### 4.1 Patient Population

We established lung cancer cohort on August 22, 2016 and have prospectively collected clinical information and tumor samples (IRB approval number: NCC2016‐0208). We also established PDCs from malignant pleural effusion samples. Among these, we retrospectively analyzed outcomes from patients who had progressed following prior EGFR‐TKI treatment (Table S1). Transcriptomic profiles from both scRNA‐seq and spatial scRNA‐seq were obtained from patients progressed to stage IV after EGFR‐TKI treatment (Table S2).

### 4.2 Sample Preparation for Single Cell RNA‐Sequencing

All details were included in the Supporting Information.

### 4.3 Target‐Seq and scRNA‐seq Analyses of ABCP‐Treated Patients

Mutation profiles were acquired from a previous study in an equal NCC PDC cohort [19]. Sample preparation and target‐seq analysis were conducted using the same protocol used in the previous study, following The Cancer Genomic Atlas Genomic Data Commons pipeline. To acquire single‐cell transcriptome profiles, samples were labeled using a BD Single‐Cell Multiplexing Kit (BD Biosciences, Franklin Lakes, NJ, USA), and cDNA libraries were constructed using the BD Rhapsody system, a BD Rhapsody Whole Transcriptome Analysis (WTA) Amplification Kit, and a BD Rhapsody cDNA Kit (BD Biosciences). Paired‐end (2 × 150 bp) sequencing was performed using an Illumina HiSeq 2500 Sequencing System (Illumina, San Diego, CA, USA). Single‐cell‐level gene expression profiles were extracted using the BD Rhapsody Analysis pipeline. All subsequent single‐cell analyses were performed using Seurat v4.3.0 [47]. In BD Rhapsody quality analysis, cells were filtered out based on the presence of double tags, mitochondrial percentage > 30%, and feature count < 200 or > 6000. Finally, gene expression profiles were acquired. To eliminate the batch effect, data were normalized for each multiplexed group using the Seurat SCTransform function, and Harmony was used [48]. The top 3000 variable features were extracted using the SelectIntegrationFeatures function. Cell clustering was performed using FindNeighbors and FindClusters, and uniform manifold approximation and projection (UMAP) was used for visualization. Differentially expressed genes (DEGs) were identified using the FindMarkers function. The top 200 DEGs in each cluster were selected based on a log2FC > 0.25 and adjusted *p* < 0.05 (Table S3). Canonical markers to identify cell types in each cluster were referred to from previous studies (Table S4). Cell type enrichment according to two groups was analyzed using the chi‐square test.

For all single‐cell analyses, trajectory trees were created using Monocle v2.26.0, and DEGs were extracted from evolving states [49]. Gene set enrichment analysis (GSEA) of the DEGs was performed using clusterProfiler v4.6.2 [50]. Reference gene sets were collected from the Hallmark, Reactome, Kyoto Encyclopedia of Genes and Genomes, WikiPathway, and Gene Ontology in MSigDB v7.5.1 [51, 52, 53, 54]. The pathway score for each cell was calculated using the AddModuleScore function. Pseudo‐bulking of the single‐cell data was performed using the average expression for each patient or cell type.

To evaluate tumor cell clusters, we performed CNV inference using inferCNV v1.14.2, using normal myeloid cells as a control [55]. CNV scores were acquired for each cell. Additionally, we analyzed whether the tumor cell clusters were close to malignant or a mixture of tumor and normal epithelial cells. For comparison with a gold standard, we collected LUAD single‐cell transcriptome (GSE131907) data acquired from tumor lung biopsies and adjacent normal tissues [22]. Epithelial‐type normal and malignant cells were extracted by cell annotation of the data. The transcriptome profiles obtained in this study were compared with GSE131907 dataset after z‐transformation rescaling. To determine the similarity of the cells identified with malignant cells, all pairwise Euclidean distances were calculated between the cells identified and GSE131907 epithelial cells.

### 4.4 Intercellular Interaction Identification

CellPhoneDB v4.0.0 was used to extract cell–cell interactions, and ligand receptors in each cell type were selected based on logFC > 0.25 and adjusted *p* < 0.05 [56]. Ligand–receptor pairs with *p* < 0.05 and rank < 0.3 were considered significant. Interaction counts between two cell types were determined from a cell–cell interaction adjacency matrix and sorted according to the ABCP response. The count network according to ABCP response was visualized using Cytoscape v3.10.0 [57]. Autocrine and paracrine ligand–receptor signaling communication among cell types was inferred and visualized using CellChat v2.1.1 [58].

### 4.5 Tissue Preparation and Spatial Transcriptome Profiling

Lung cancer formalin‐fixed paraffin‐embedded (FFPE) tissue sections were acquired from patients with EGFR‐mutant advanced NSCLC who had progressed after osimertinib treatment. We followed the Visium CytAssist Spatial Gene Expression for FFPE workflow for sectioning and RNA quality assessment (DV200 ≥ 30%). Tissue sections were deparaffinized, stained with hematoxylin and eosin, imaged, and decross‐linked, immediately followed by probe hybridization using a Visium Spatial Gene Expression for FFPE reagent kit (10X Genomics). The probe pairs hybridize with their target genes and are then ligated to one another. The single‐stranded ligation products are released from the tissue to a capture area on the Visium slide in the CytAssist instrument. Once the ligation products were captured, the slides were removed from the instrument, and probes were extended by the addition of a unique molecular identifier, Spatial Barcode, and Partial read 1.

To extract gene expression profiles, we used the Space Ranger v2.1.1 (10X Genomics) pipeline for FASTQ file generation from raw sequencing data. Visium spatial expression libraries were analyzed using the “count” command. Image processing proceeded in two stages. First, we used the fiducial alignment grid of the tissue image to determine the orientation and position of the input image. Next, we determined the region covered by the tissue on the slide. Sequencing reads were aligned to the GRCh38 reference genome using STAR (v2.5.1b) [59]. Normalization, dimension reduction, clustering, and DEG testing were performed using Seurat. After identifying variable features and anchors according to the “moransi” method using the SpatiallyVariableFeatures and FindTransferAnchors functions, respectively, cell type prediction was performed using the TransferData function in Seurat by referencing the previously identified cell types. Signature and pathway scores were calculated using the AddModuleScore function. Cell types were annotated to each cluster based on the scores using previously assessed ABCP patient cell type DEGs. Certain cell types unidentified in MPE samples were annotated using canonical markers (Table S4). A cell–cell communication network was established and investigated using CellChat.

### 4.6 Multiplex IHC Staining of FFPE Tissues

All details were included in the Supporting Information.

### 4.7 Multispectral Imaging and Analysis

All details were included in the Supporting Information.

### 4.8 Cellular Mechanism Evaluation According to Tumor Progression After TKI Treatment

All details were included in the Supporting Information.

### 4.9 Clinical Outcome Investigation Using Meta‐Transcriptome Analysis of Multiple Cohorts

All details were included in the Supporting Information.

### 4.10 VEGFA/C Global Interaction Network Analysis

All details were included in the Supporting Information.

### 4.11 In Vitro and In Vivo VEGF Signaling Analyses

All details were included in the Supporting Information.

## Author Contributions

J.H. and C.P. developed the study concept and experimental design. J.Y.H., G.K.L., B.C.A., and Y.L. contributed to the management of ABCP therapy and NCC cohorts and the collection of patient clinical information. S.H., N.Y., and C.P. performed the computational analyses. J.Y.C. and J.H.K. performed the *i*n vitro and in vivo experiments. H.S., B.R.S., M.H., and S.K. collected the patient samples. J.H.K., C.P., and J.Y.H. wrote and revised the manuscript. All authors approved the final manuscript.

## Conflicts of Interest

The authors declare no conflicts of interest.

## Ethics Statement

This study was approved by the Institutional Review Board (IRB), and all patients provided informed consent. (approval No. NCC2016‐0208).

## Supporting information



Table S1. Clinical profile of patients to receive ABCP treatment.

**Figure S1. Tumor cell cluster analysis**. A) Euclidean distance heatmap of cell type expression profile between our tumor subcluster and GSE131907 epithelial subcluster. B) Heatmap of top‐ranked differentially expressed genes for tumor subclusters.
**Figure S2. Immune cell cluster analysis**. A) Analysis of myeloid cell type for subcluster identification, its cell proportions, and its canonical markers. Pathways for subclusters were further assessed by gene set enrichment analysis (GSEA). B) Analysis of lymphoid cell type for subcluster identification, its cell proportions, and its canonical markers. Pathways were investigated by GSEA. C) Macrophage subclusters’ cell proportions according to ABCP response, and its canonical markers.
**Figure S3. Additional spatial transcriptome analysis**. Capture area was 6.5 x 6.5 mm. A‐B are results of Slide1. C‐G are associated with Slide2. A) Cell score distribution on Slide1 according to tumor, stromal, lymphoid, and myeloid scores. B) Heatmap of marker expression to identify the cell type. C) Cell type proportion bar plot and its distribution in Slide2. D) Cell lineage scores for identified cell types. F) Heatmap of expression and scores of VEGF genes and pathways for cell types of Slide2. E) VEGFA and VEGF expression on Slide2 and circular plots of its cell–cell interactions. G) Bar plots of odds ratios (ORs) for *VEGFA* and *VEGFC* expression according to cell types.
**Figure S4. Heatmap of gene expression profile according to cell types and therapeutic groups**. TKI treatment groups were classified into three types: TN, treatment‐naïve (blue); RD, residual disease (yellow‐green), PD, progressive disease (orange).
**Figure S5. Heatmap of gene–gene interaction network module analysis from *VEGFA‐* and *VEGFC*‐regulated networks**. Pathways for each module were extracted by gene set enrichment analysis. Pathways categorized as ‘Common’ were detected in both *VEGFA* and *VEGFC* networks. The dominant mechanisms of *VEGFA* modules (VEGFA_0‐VEGFA_7) and *VEGFC* (VEGFC_0‐VEGFC_7) are highlighted for each case.
**Figure 6. Combinational knockdown of *VEGFC* and its receptor (*KDR* and *NRP2*) efficiently enhance the anti‐proliferation and anti‐angiogenesis effects of cabozantinib *in vitro***. A) qPCR results demonstrate the relative mRNA expression of *VEGFC*, *KDR*, *NRP2*, *VEGFA*, and *FLT4* in diverse lung cancer cell lines (*n* = 3). B) IC_50_ table of lung cancer cell lines exposed to three VEGF inhibitors. Three cell lines highlighted in red exhibited higher IC_50_ values associated with increased expression of *VEGFC*, *KDR*, and *NRP2*. C) Bar graphs depict qPCR results (*n* = 3) confirming the efficiency of target gene silencing. D‐E) MTT assay results show the relative cell proliferation ratio following cabozantinib treatment with or without specific target gene knockdown in H1573 (D) and H2279 (E) cell lines. The orange bars below each graph indicate cabozantinib treatment (*n* = 3). F) qPCR results demonstrate the relative mRNA expression of *YAP1* (left) and *TAZ* (right) following target gene silencing with or without cabozantinib treatment (marked by an orange bar). G) In vitro angiogenesis assay demonstrates the relative change in capillary‐like tubular structures of HUVECs on Matrigel by conditioned media (CM) treatment. CMs were harvested from H2279 cells following cabozantinib treatment with or without target gene silencing. (Left) Phase contrast microscopic images of capillary‐like tubular structures on Matrigel. Magnification, x100. (Right) Graph bars show the tube length of HUVECs. *P<0.05, **P<0.01, ***P<0.001, ****P<0.0001, one‐way ANOVA, followed by Tukey's multiple‐comparison test.

Supporting information

## Data Availability

All gene expression profiling data generated in this study were deposited in the Gene Expression Omnibus (ABCP: scRNA‐seq GSE233203, spatial scRNA‐seq GSE267960; NCC PDC: GSE165611, GSE229535, and PRJNA694788).
