# Supplementary material for: VEGF Signal Complexity Confers Resistance to Atezolizumab, Bevacizumab, Carboplatin, and Paclitaxel in EGFR‐Tyrosine Kinase Inhibitor‐Resistant Non‐Small Cell Lung Cancer
Source: MedComm (2020). 2025 Aug 19;6(9):e70335. doi: 10.1002/mco2.70335 (PMC12365383; doi:10.1002/mco2.70335)
Supplement: Supplementary file 2 — Figure S1. Tumor cell cluster analysis. A) Euclidean distance heatmap of cell type expression profile between our tumor subcluster and GSE131907 epithelial subcluster. B) Heatmap of top‐ranked differentially expressed genes for tumor subclusters. Figure S2. Immune cell cluster analysis. A) Analysis of myeloid cell type for subcluster identification, its cell proportions, and its canonical markers. Pathways for subclusters were further assessed by gene set enrichment analysis (GSEA). B) Analysis of lymphoid cell type for subcluster identification, its cell proportions, and its canonical markers. Pathways were investigated by GSEA. C) Macrophage subclusters’ cell proportions according to ABCP response, and its canonical markers. Figure S3. Additional spatial transcriptome analysis. Capture area was 6.5 x 6.5 mm. A‐B are results of Slide1. C‐G are associated with Slide2. A) Cell score distribution on Slide1 according to tumor, stromal, lymphoid, and myeloid scores. B) Heatmap of marker expression to identify the cell type. C) Cell type proportion bar plot and its distribution in Slide2. D) Cell lineage scores for identified cell types. F) Heatmap of expression and scores of VEGF genes and pathways for cell types of Slide2. E) VEGFA and VEGF expression on Slide2 and circular plots of its cell–cell interactions. G) Bar plots of odds ratios (ORs) for VEGFA and VEGFC expression according to cell types. Figure S4. Heatmap of gene expression profile according to cell types and therapeutic groups. TKI treatment groups were classified into three types: TN, treatment‐naïve (blue); RD, residual disease (yellow‐green), PD, progressive disease (orange). Figure S5. Heatmap of gene–gene interaction network module analysis from VEGFA‐ and VEGFC‐regulated networks. Pathways for each module were extracted by gene set enrichment analysis. Pathways categorized as ‘Common’ were detected in both VEGFA and VEGFC networks. The dominant mechanisms of VEGFA modules (VEGFA_0‐VEGFA_7) and VEGFC [file MCO2-6-e70335-s003.docx]

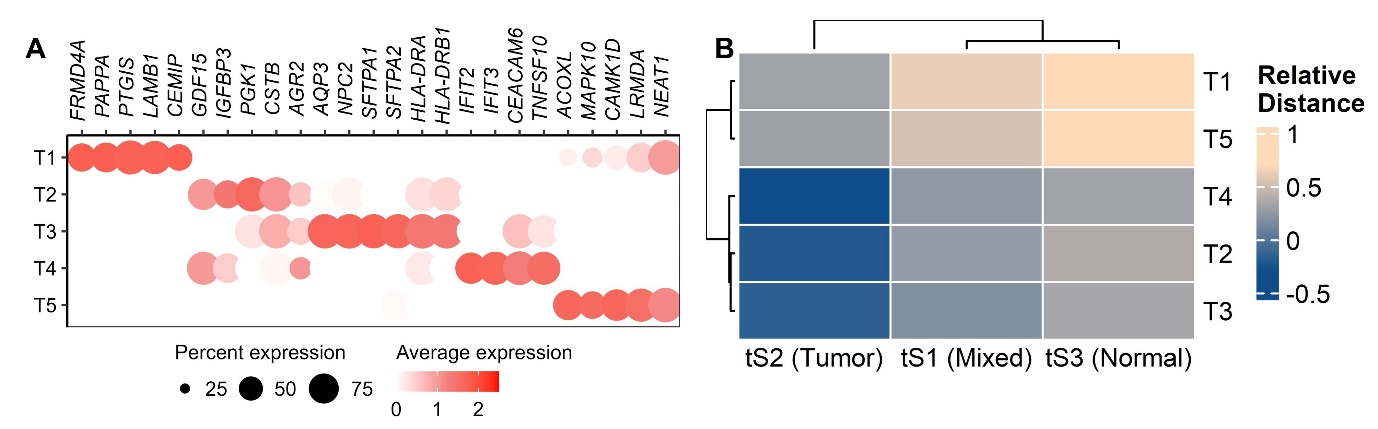


**Supplementary Fig. S1. Tumor cell cluster analysis.** A) Euclidean distance heatmap of cell type expression profile between our tumor subcluster and GSE131907 epithelial subcluster. B) Heatmap of top-ranked differentially expressed genes for tumor subclusters.

**
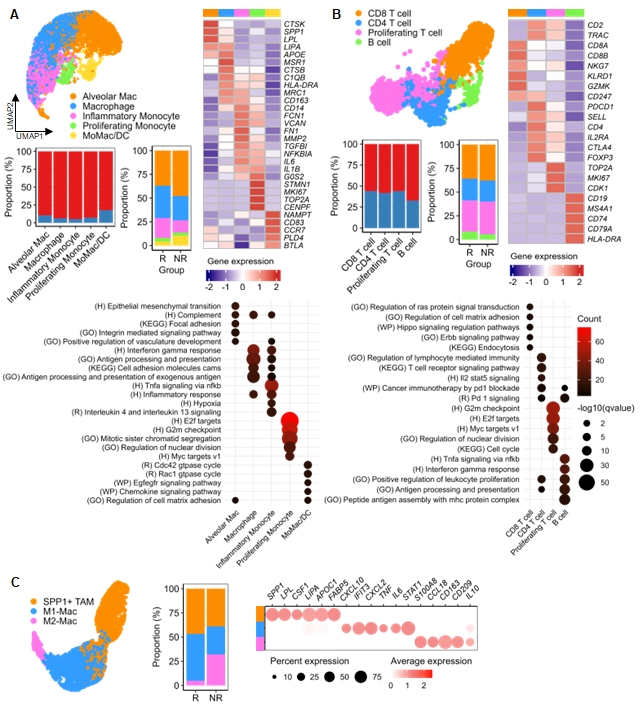
**

**Supplementary Fig. S2. Immune cell cluster analysis.** A) Analysis of myeloid cell type for subcluster identification, its cell proportions, and its canonical markers. Pathways for subclusters were further assessed by gene set enrichment analysis (GSEA). B) Analysis of lymphoid cell type for subcluster identification, its cell proportions, and its canonical markers. Pathways were investigated by GSEA. C) Macrophage subclusters’ cell proportions according to ABCP response, and its canonical markers.

**
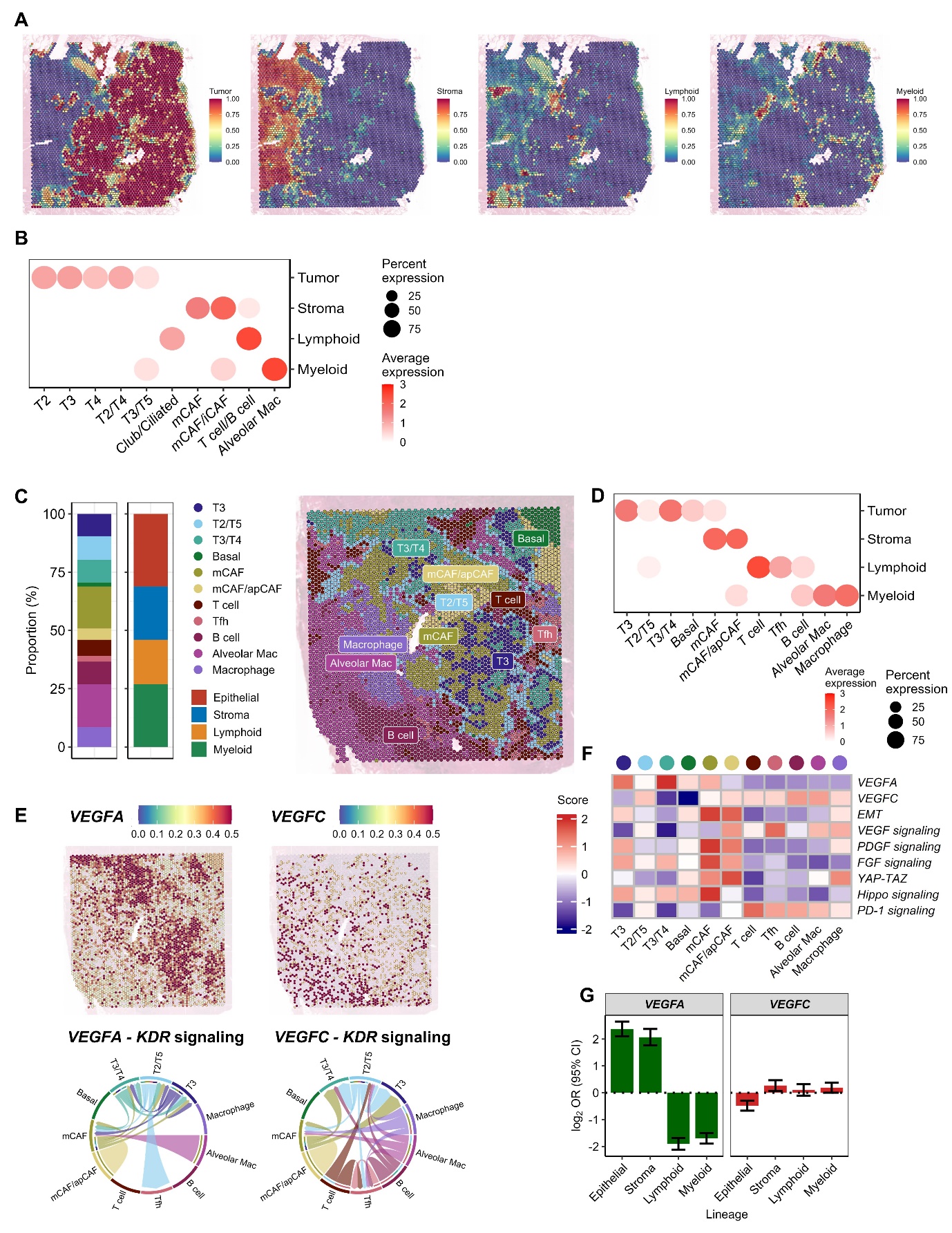
**

**Supplementary Fig. S3. Additional spatial transcriptome analysis.** Capture area was 6.5 x 6.5 mm. A-B are results of Slide1. C-G are associated with Slide2. A) Cell score distribution on Slide1 according to tumor, stromal, lymphoid, and myeloid scores. B) Heatmap of marker expression to identify the cell type. C) Cell type proportion bar plot and its distribution in Slide2. D) Cell lineage scores for identified cell types. F) Heatmap of expression and scores of VEGF genes and pathways for cell types of Slide2. E) VEGFA and VEGF expression on Slide2 and circular plots of its cell–cell interactions. G) Bar plots of odds ratios (ORs) for *VEGFA* and *VEGFC* expression according to cell types.

**
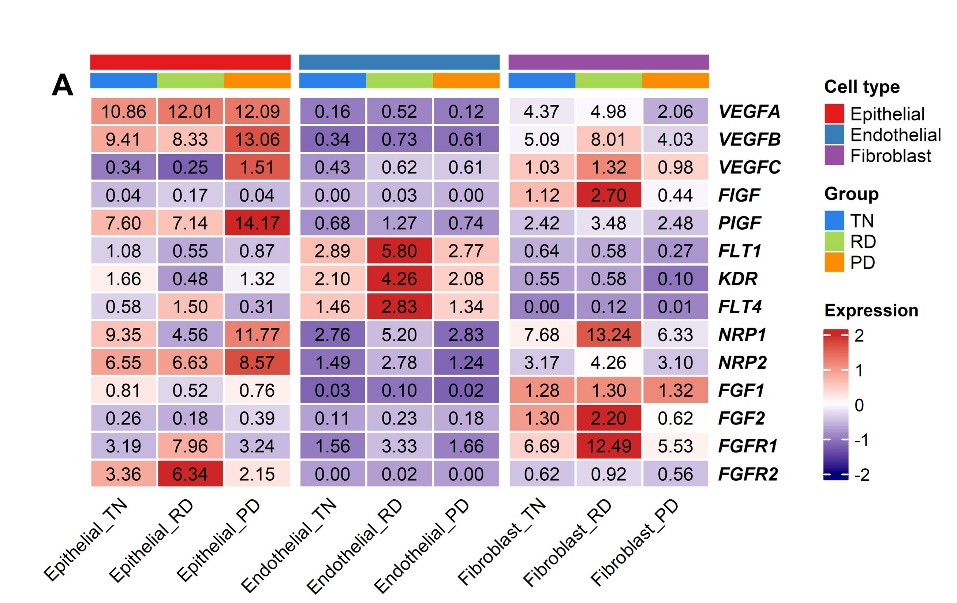
**

**Supplementary Fig. S4. Heatmap of gene expression profile according to cell types and therapeutic groups.** TKI treatment groups were classified into three types: TN, treatment-naïve (blue); RD, residual disease (yellow-green), PD, progressive disease (orange).

**
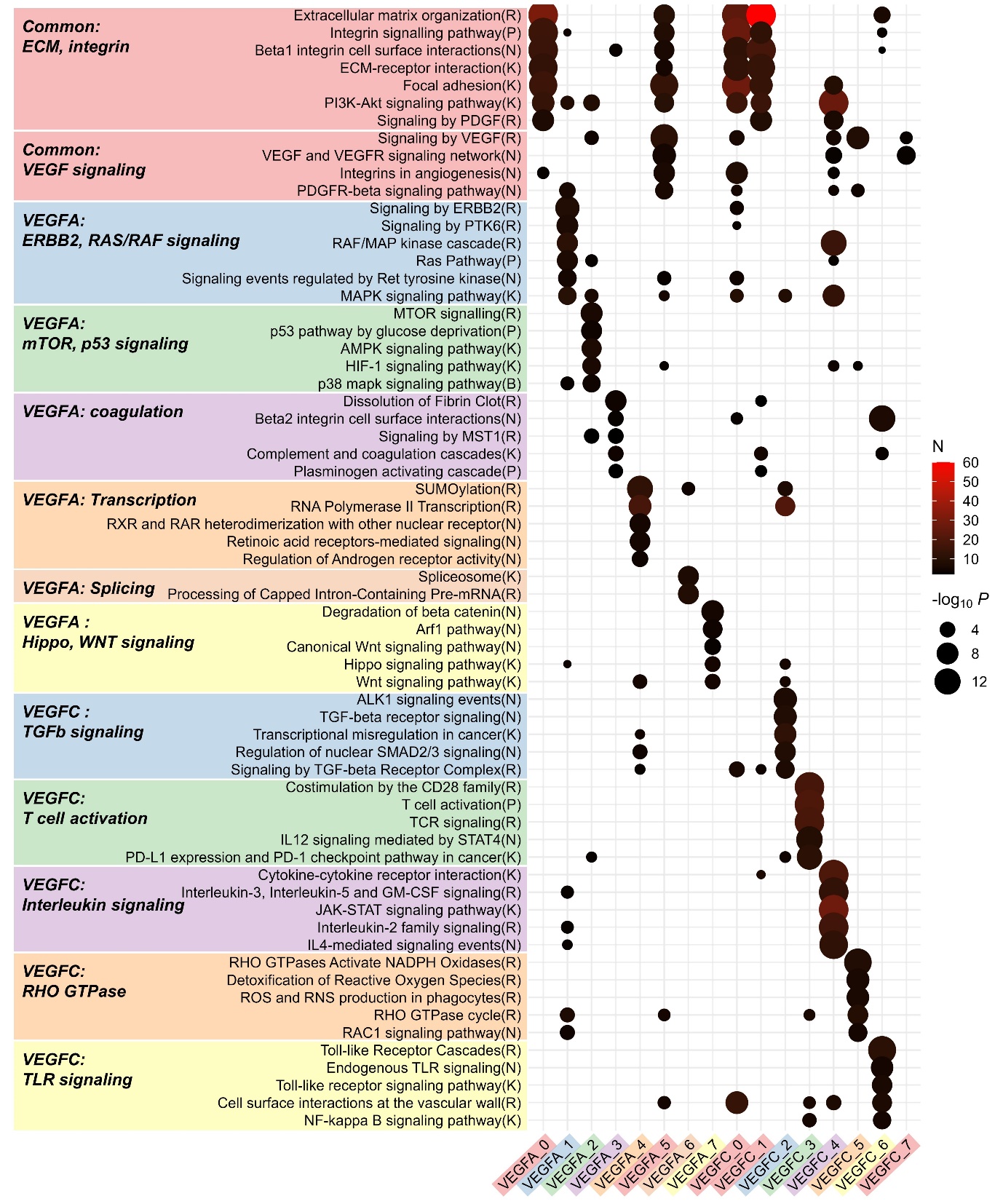
**

**Supplementary Fig. S5. Heatmap of gene–gene interaction network module analysis from *VEGFA-* and *VEGFC*-regulated networks.** Pathways for each module were extracted by gene set enrichment analysis. Pathways categorized as ‘Common’ were detected in both *VEGFA* and *VEGFC* networks. The dominant mechanisms of *VEGFA* modules (VEGFA_0-VEGFA_7) and *VEGFC* (VEGFC_0-VEGFC_7) are highlighted for each case.

**
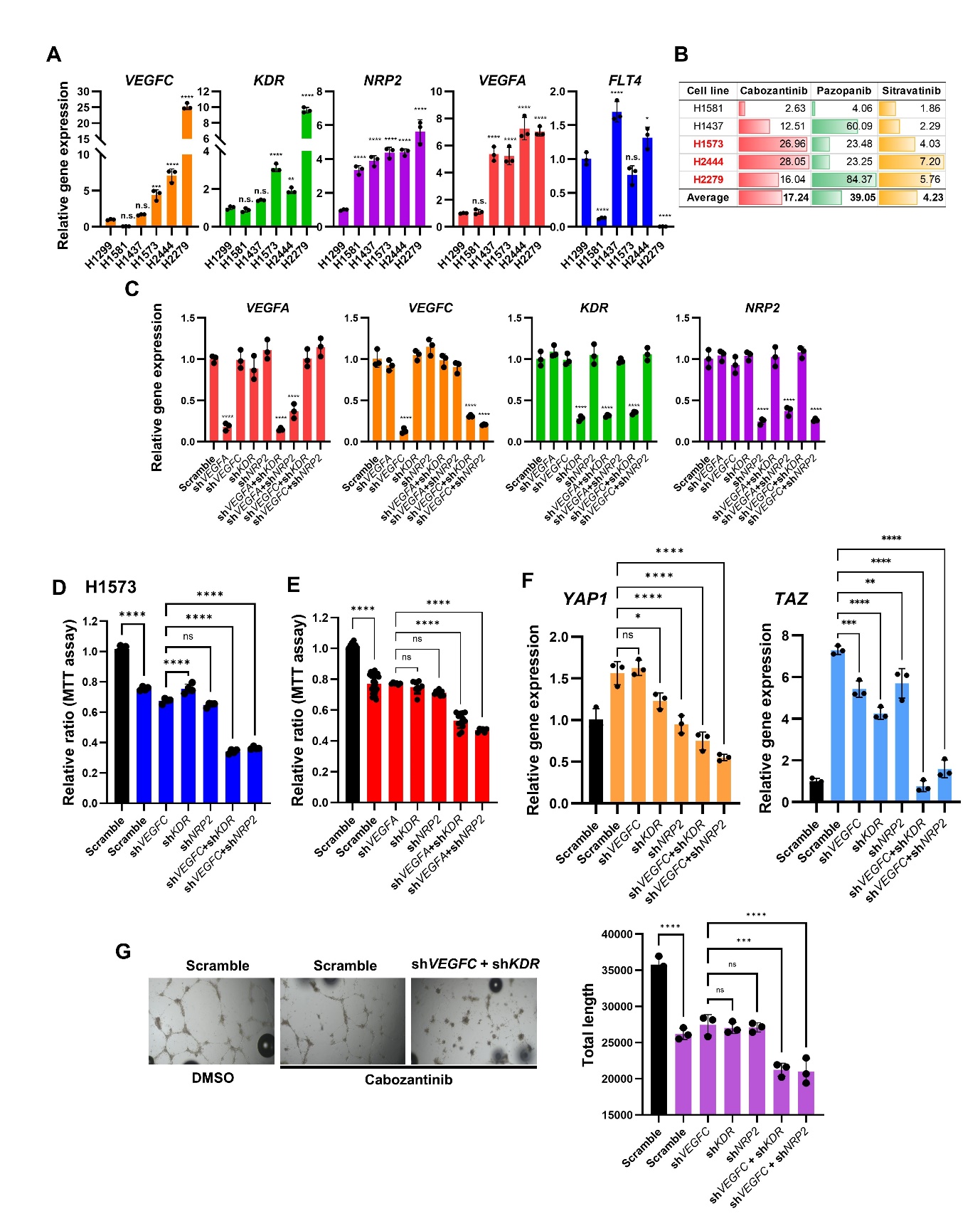
**

**Supplementary Fig. 6. Combinational knockdown of *VEGFC* and its receptor (*KDR* and *NRP2*) efficiently enhance the anti-proliferation and anti-angiogenesis effects of cabozantinib *in vitro*.** A) qPCR results demonstrate the relative mRNA expression of *VEGFC*, *KDR*, *NRP2*, *VEGFA*, and *FLT4* in diverse lung cancer cell lines (*n*=3). B) IC_50_ table of lung cancer cell lines exposed to three VEGF inhibitors. Three cell lines highlighted in red exhibited higher IC_50_ values associated with increased expression of *VEGFC*, *KDR*, and *NRP2*. C) Bar graphs depict qPCR results (*n*=3) confirming the efficiency of target gene silencing. D-E) MTT assay results show the relative cell proliferation ratio following cabozantinib treatment with or without specific target gene knockdown in H1573 (D) and H2279 (E) cell lines. The orange bars below each graph indicate cabozantinib treatment (*n*=3). F) qPCR results demonstrate the relative mRNA expression of *YAP1* (left) and *TAZ* (right) following target gene silencing with or without cabozantinib treatment (marked by an orange bar). G) *In vitro* angiogenesis assay demonstrates the relative change in capillary-like tubular structures of HUVECs on Matrigel by conditioned media (CM) treatment. CMs were harvested from H2279 cells following cabozantinib treatment with or without target gene silencing. (Left) Phase contrast microscopic images of capillary-like tubular structures on Matrigel. Magnification, x100. (Right) Graph bars show the tube length of HUVECs.

**P*<0.05, ***P*<0.01, ****P*<0.001, *****P*<0.0001, one-way ANOVA, followed by Tukey’s multiple-comparison test.
