# Supplementary material for: VEGF Signal Complexity Confers Resistance to Atezolizumab, Bevacizumab, Carboplatin, and Paclitaxel in EGFR‐Tyrosine Kinase Inhibitor‐Resistant Non‐Small Cell Lung Cancer
Source: MedComm (2020). 2025 Aug 19;6(9):e70335. doi: 10.1002/mco2.70335 (PMC12365383; doi:10.1002/mco2.70335)
Supplement: Supplementary file 3 — Supporting information [file MCO2-6-e70335-s001.docx]

**Supplementary Information**

***Sample collection, and data analysis***

***4.2 Sample preparation for single cell RNA-sequencing***

All samples used for scRNA-seq were collected from pleural effusions obtained from patients with histologically proven NSCLC, following institutional review board approval (approval No. NCC2016-0208; Table S1). Cells were isolated from pleural effusions by density gradient centrifugation using Lymphocyte Separation Medium (LSM, #091692249; MP Biomedicals, Solon, OH, USA) following the manufacturer’s instructions. After washing, the cells were cultured in AR-5 medium (5% fetal bovine serum [FBS], 1X GlutaMAX [Thermo Fisher Scientific, Waltham, MA, USA), 1X Insulin-Transferrin-Selenium [Thermo Fisher Scientific], 1% penicillin/streptomycin, 50 nM hydrocortisone, 1 mM sodium pyruvate, and 1 ng/mL EGF in Roswell Park Memorial Institute [RPMI] 1640 medium) at 37°C in a 5% CO_2_ atmosphere. The medium was changed carefully every 2–3 days until the cells were stabilized in the flask. The cells were suspended in a freezing medium (10% dimethylsulfoxide [DMSO in FBS] and cryopreserved in liquid N_2_ until analysis.

Clinical profiles were obtained from TKI-resistant patients prior to ABCP therapy, mono-immunotherapy (immune oncology drugs; IO), or chemotherapy (pemetrexed and carboplatin, PemC) (Table S2). The proportion of responders according to the treatment group was tested using Fisher’s exact test. For statistical analysis of other characteristics, we used Fisher’s exact test for binary variables and analysis of variance (ANOVA) for continuous variables. Cox-hazard regression analysis and Kaplan–Meier survival analysis were conducted using the R packages (survival v3.4-0 and survminer v0.4.9).

***4.6 Multiplex IHC staining of FFPE tissues***

Multiplex IHC staining, scanning, and analysis were performed using a prismCDX instrument (prismCDX, Gyeonggi, Korea). FFPE blocks were cut into 4-μm sections. The sections were dried in an oven at 60°C, followed by multiplex immunofluorescence staining using a Leica Bond Rx™ Automated Stainer (Leica Biosystems). Briefly, the slides were dewaxed using Leica Bond Dewax solution (#AR9222; Leica Biosystems), followed by antigen retrieval using Bond Epitope Retrieval 2 (#AR9640; Leica Biosystems). Staining proceeded in sequential rounds of blocking (#C0103; TheraNovis) followed by primary antibody and secondary antibody (#C0105; TheraNovis) incubations. Antigen visualization was accomplished using Astra-dye (TheraNovis) after the slide was treated with Bond Epitope Retrieval 1 (#AR9961; Leica Biosystems). Nuclei were counterstained with DAPI (62248; Thermo Scientific) after the last round of antigen retrieval. The sections were covered with ProLong Gold antifade reagent (P36930; Invitrogen) and a coverslip. The antibodies and fluorophores used are listed in Table S5.

***4.7 Multispectral imaging and analysis***

Multiplex-stained slides were scanned using a PhenoImager HT (Akoya Biosciences) at a magnification of 20×. Representative images for training were selected using Phenochart Whole Slide Viewer (v1.1.0, Akoya Biosciences), and an algorithm was created using the inForm Tissue Analysis software (v2.6, Akoya Biosciences). Multispectral images were unmixed using the spectral library in inForm. Each cell was segmented based on DAPI staining and phenotyped based on the expression compartment and intensity of each marker. After designating the region to be analyzed on the tissue slide, the same algorithm was applied in batch mode for analysis. The exported data were consolidated and analyzed in R studio (v4.2.1) using the phenoptr and phenoptrReport packages (Akoya Biosciences).

***4.8 Cellular mechanism evaluation according to tumor progression after TKI treatment***

To validate our results, we collected another single-cell transcriptome dataset (PRJNA591860) acquired from surgical resection samples of patients with NSCLC.^1^ The patients were grouped according to their treatment response into TN, RD during initial treatment, and PD. Cell types were assigned according to a previous study.^1^ The proportions of cells expressing genes of interest by cell type and response or sample origin were determined for the GSE131907 and PRJNA591860 datasets and compared using Fisher’s exact test. Seven non-response pathway signals obtained from ABCP transcriptome analysis (epithelial-to-mesenchymal transition [EMT], VEGF, platelet-derived growth factor [PDGF], fibroblast growth factor [FGF], Hippo, programmed cell death 1 [PD-1], and YAP-TAZ signaling) were interrogated in PRJNA591860 using the AddModuleScore function in Seurat. The pathway scores or gene expression at the single-cell level were transformed to representative values at the cell type-level for each patient or patient-level through the pseudo-bulking aggregation method by summing all measures. We investigated the correlations between these pathways and VEGFA/VEGFC-regulatory pathways for all cell types.

***4.9 Clinical outcome investigation using meta-transcriptome analysis of multiple cohorts***

We validated our findings using lung cancer transcriptome datasets from large-scale and multiple cohorts. NCC cohort profiles (NCC; *n* = 95, accession IDs GSE165611, GSE229535, and PRJNA694788; Table S6) from refractory lung cancer patients were collected. Additionally, six public microarray- and RNA-seq-based NSCLC transcriptome datasets (META1460; *n* = 1460) were collected from the Gene Expression Omnibus and The Cancer Genome Atlas (Table S7). All META1460 datasets were rescaled to log2 scale and merged after batch removal using the ComBat function in the sva v3.46.0 package.^2,3^

To evaluate clinical outcomes and distinct global mechanisms induced by VEGFA and VEGFC, NCC patients were classified into baseline, responder, and non-responder groups based on their TKI treatment history. Using gene set variation analysis, we determined regulation scores across gene signatures of CAF subtypes and TME signaling pathways inferred from our single-cell transcriptome analysis.^4^ Patients were divided into high (upper median) and low (lower median) *VEGFA* and *VEGFC* expression groups. Pathway score differences between the high and low expression groups were tested using the Wilcoxon signed-rank test. Survival analysis based on these datasets was conducted as mentioned above.

***4.10 VEGFA/C global interaction network analysis***

To uncover the distinct regulatory mechanisms of *VEGFA* and *VEGFC*, we analyzed co-expression networks using the META1460 transcriptomes. First, we generated a global reference gene–gene interaction network (10,647 nodes and 54,037 edges) using multiple databases (DIP, BIND, HPRD, and REACTOME).^5–8^ To rank network genes regulated by VEGFA or VEGFC, we calculated one-sided *p*-values using the limma test between *VEGFA-* and *VEGFC*-high and -low expression groups. The *p*-values were fitted using the beta-uniform mixture model, and node scores were derived based on false discovery rates <0.001 and <1e-10 for *VEGFA* and *VEGFC*, respectively. Maximum-score subnetworks enriched with positive-scored nodes were extracted for *VEGFA* and *VEGFC* using the Heinz algorithm in BioNet v1.58.0.^9^ Next, we clustered the subnetworks to identify regulatory modules and investigated the biological functions of each module using Cytoscape ReactomeFIViz.^10^ Finally, we obtained *VEGFA-* and *VEGFC*-regulated interaction networks and the associated mechanisms.

***In vitro* and *in vivo* VEGF signaling analyses**

***4.11.1 Cell lines***

H1437 (#CRL-5872; ATCC, Manassas, VA, USA), H1573 (#CRL-5877; ATCC), H2444 (#CRL-5945; ATCC), H1581 (#91581; KCLB, Seoul, Korea), and H2279 (#72279; KCLB) cells were maintained in RPMI medium (Cat# RPM-002; SolBio), supplemented with 10% FBS (Cat# FP-0500-A; Atlas) and 100 U/mL penicillin/streptomycin (Cat# LS202-02; Welgene). Human umbilical vein endothelial cells (HUVECs) were purchased from Life Technologies and cultured in Medium 200PRF (M-200PRF-500; Invitrogen) supplemented with low-serum growth supplement (S-003-10; Invitrogen). All cell lines were maintained in a humidified incubator at 37°C with 5% CO_2_.

***4.11.2 Plasmid construction***

Lentiviral shRNA constructs targeting *VEGFC* (5′-GCAAGACGTTATTTGAAAT-3′), *VEGFA* (5′-AGGGCAGAATCATCACGAAGT-3′), *NRP2* (5′-CGACTGCAAGTATGACTTTAT-3′), or *KDR* (5′-GCGGCTACCAGTCCGGATA-3′) and control scrambled shRNA (5′-CCTAAGGTTAAGTCGCCCTCG-3′) were designed and constructed at VectorBuilder. All plasmids were verified by sequencing.

***4.11.3 Lentivirus production***

Recombinant lentiviruses were produced by cotransfecting lentiviral shRNA vectors along with three packaging vectors (pMDLg/pRRE, pRSV-REV, and pVSVG) into HEK293 FT cells using polyethylenimine (Sigma–Aldrich). Lentiviral supernatants were collected 48 h after transfection and clarified by filtration before use. Lentiviruses were concentrated using ultracentrifugation in an Optima XE-90K centrifuge (Beckman) with an SW32TI rotor (Beckman) at 19,500 rpm, 20°C for 2 h. Supernatants were removed, and virus pellets were resuspended in phosphate-buffered saline (PBS).

***4.11.4 Quantitative reverse transcription (RT-q)PCR***

Total RNA was isolated from cells using an Axen Total RNA kit (Macrogen) and treated with RNase-free DNase I (Macrogen). RT was performed using 0.5–1 μg of total RNA, random hexamer, and a SuperScript III First Strand Synthesis Kit (Thermo Fisher Scientific). qPCRs were run in triplicate using Axen qPCR Master Mix (Macrogen) on a LightCycler 96 Real-Time PCR System (Roche). The thermal cycling program was as follows: initial denaturation at 95°C for 10 min, followed by 40 cycles of denaturation at 95°C for 10 s, and annealing at 60°C for 30 s. Gene expression levels were normalized to *GAPDH* expression. The PCR primers used are listed in Table S8.

***4.11.5 Cell proliferation assay***

Cell proliferation was measured using the 3-[4,5-dimethylthiazol-2-yl]-2,5 diphenyl tetrazolium bromide (MTT) assay. Cells (1 × 10^4^) were seeded in 96-well plates in 150 µL of medium per well. After 7 days, 20 µL of MTT solution (Thiazolyl Blue Tetrazolium Bromide; 5 mg/mL; Sigma–Aldrich) was added into each well and the plate was incubated in the dark for 4 h. Next, 150 µL of solubilization solution (0.1% NP-40 and 1 N HCl in anhydrous isopropanol) was added to dissolve the formazan crystals. The absorbance at 570 nm was measured using a SPECTROstar Nano Microplate Reader (BMG Labtech).

***4.11.6 In vitro angiogenesis assay***

Cancer cell lines were cultured in complete serum-supplemented media until 90% confluence was achieved. Cells were washed with 1× PBS and then starved by incubation in serum-free medium for 12 h prior to collection of the medium. Cell-free supernatant (conditioned medium) was collected for HUVEC assays. HUVECs in each conditioned medium were seeded in a 24-well plate coated with growth factor-reduced Matrigel (#356234, BD Biosciences). Matrigel was prepared at a 1:2 ratio with one part Matrigel and two parts Medium 200PRF. Tube formation was examined at different time points, and images were acquired with an inverted microscope (CKX53 Cell Culture Microscope; Olympus). Tubes and branching points were quantified using Fiji software (v1.54j).

***4.11.7 Nude mouse lung cancer xenograft model***

All animal procedures were performed in accordance with a protocol approved by the Institutional Animal Care and Use Committee (#NCC-23-952-001) of the National Cancer Center Research Institute. Nude mice (BALB/cAnN.Crj-nu/nu) were obtained from Orient. H2444 human lung cancer cells (1 × 10^6^) were resuspended in 50 µL of PBS, mixed with 50 µL of Matrigel, and implanted subcutaneously into the right flanks of 6–7-week-old mice. To distinguish individual differences among mice, six mice were randomly chosen for four groups: bevacizumab, ramucirumab, bevacizumab plus ramucirumab, or human IgG control. Forty-nine days after cell injection, the tumor was dissected and weighed. Tumor diameters were measured using a caliper.

***4.11.8 Statistical analysis***

Data were assessed for a normal distribution (Ryan–Joyner). Normally distributed data were analyzed using a two-tailed Student’s *t*-test for two groups or one-way ANOVA followed by Tukey’s multiple comparison tests for multiple groups. Non-normally distributed data were analyzed using the Mann–Whitney test for two groups or Kruskal–Wallis followed by Dunn’s multiple comparisons test for multiple groups. Statistical significance was set to *p* < 0.05.

**References**

1 Maynard A, McCoach CE, Rotow JK, Harris L, Haderk F, Kerr DL *et al.* Therapy-Induced Evolution of Human Lung Cancer Revealed by Single-Cell RNA Sequencing. *Cell* 2020; **182**: 1232-1251.e22.

2 Zhang Y, Parmigiani G, Johnson WE. ComBat-seq: Batch effect adjustment for RNA-seq count data. *NAR Genom Bioinform* 2020; **2**: lqaa078.

3 Johnson WE, Li C, Rabinovic A. Adjusting batch effects in microarray expression data using empirical Bayes methods. *Biostatistics* 2007; **8**: 118–127.

4 Hänzelmann S, Castelo R, Guinney J. GSVA: Gene set variation analysis for microarray and RNA-Seq data. *BMC Bioinformatics* 2013; **14**: 7.

5 Xenarios I, Rice DW, Salwinski L, Baron MK, Marcotte EM, Eisenberg D. DIP: The database of interacting proteins. *Nucleic Acids Res* 2000; **28**: 289–291.

6 Bader GD, Betel D, Hogue CW V. BIND: The biomolecular interaction network database. *Nucleic Acids Res* 2003; **31**: 248–250.

7 Keshava Prasad TS, Goel R, Kandasamy K, Keerthikumar S, Kumar S, Mathivanan S *et al.* Human protein reference database-2009 update. *Nucleic Acids Res* 2009; **37**: D767–D772.

8 Jassal B, Matthews L, Viteri G, Gong C, Lorente P, Fabregat A *et al.* The reactome pathway knowledgebase. *Nucleic Acids Res* 2020; **48**: D498–D503.

9 Beisser D, Klau GW, Dandekar T, Müller T, Dittrich MT. BioNet: An R-Package for the functional analysis of biological networks. *Bioinformatics* 2010; **26**: 1129–1130.

10 Wu G, Dawson E, Duong A, Haw R, Stein L. ReactomeFIViz: A cytoscape app for pathway and network-based data analysis. *F1000Res* 2014; **3**: 146.
